# DES-Based Strategies for Agri-Food By-Product Valorization and Emerging DES–Lipase Systems for Mycotoxin Mitigation

**DOI:** 10.3390/molecules31213815

**Published:** 2026-10-22

**Authors:** Joana Santos, Armando Venâncio, Carla Silva

**Affiliations:** 1 Centre of Biological Engineering, University of Minho, Campus de Gualtar, 4710-057 Braga, Portugal; joanasantos587@hotmail.com (J.S.); avenan@deqb.uminho.pt (A.V.); 2 LABBELS—Associate Laboratory, 4710-057 Braga, Portugal

**Keywords:** deep eutectic solvents, green extraction, bioactive compounds, enzymatic degradation, food safety, circular bioeconomy

## Abstract

Mycotoxins remain a major threat to food and feed safety due to their high stability and toxicity. Enzymatic degradation has emerged as a potential strategy to transform mycotoxins or reduce their measured parent concentration, while confirmation of detoxification requires identification and toxicological assessment of the resulting products. Lipases have shown potential for degrading relevant mycotoxins, including ochratoxin A (OTA), patulin, and aflatoxin M1; however, their efficiency is strongly influenced by the reaction medium, which affects enzyme stability and substrate accessibility. Recent evidence demonstrated a 94% reduction in OTA concentration using porcine pancreatic lipase in a glycerol-based DES-containing model system after 4 h, while up to approximately 93% degradation was achieved in grape pomace extract after 24 h under optimized enzyme concentrations. Deep eutectic solvents (DESs) have emerged as tunable solvent systems that have been widely explored as greener alternatives to conventional solvents, with particular potential to modulate lipase performance and have also been widely explored for the extraction of bioactive compounds from agri-food by-products. However, direct evidence of lipase-mediated mycotoxin degradation in DES-containing systems remains limited, and broader applications involving other mycotoxins, DES formulations, and engineered lipases remain largely unexplored. These complementary applications are particularly relevant within the circular bioeconomy, where residues such as grape pomace, apple pomace, and tomato by-products are valuable sources of phenolic compounds but may also be affected by mycotoxin contamination. Enzyme immobilization and chemical modification further offer complementary strategies to improve lipase stability, activity, and reusability in DES-containing systems. This review critically discusses the role of DESs in lipase-mediated mycotoxin degradation, their compatibility with free and immobilized or chemically modified lipases, and their application in the safe extraction and valorization of contaminated agri-food by-products. Key gaps include analytical validation and the identification and toxicological assessment of transformation products.

## 1. Introduction

**Figure 1:**
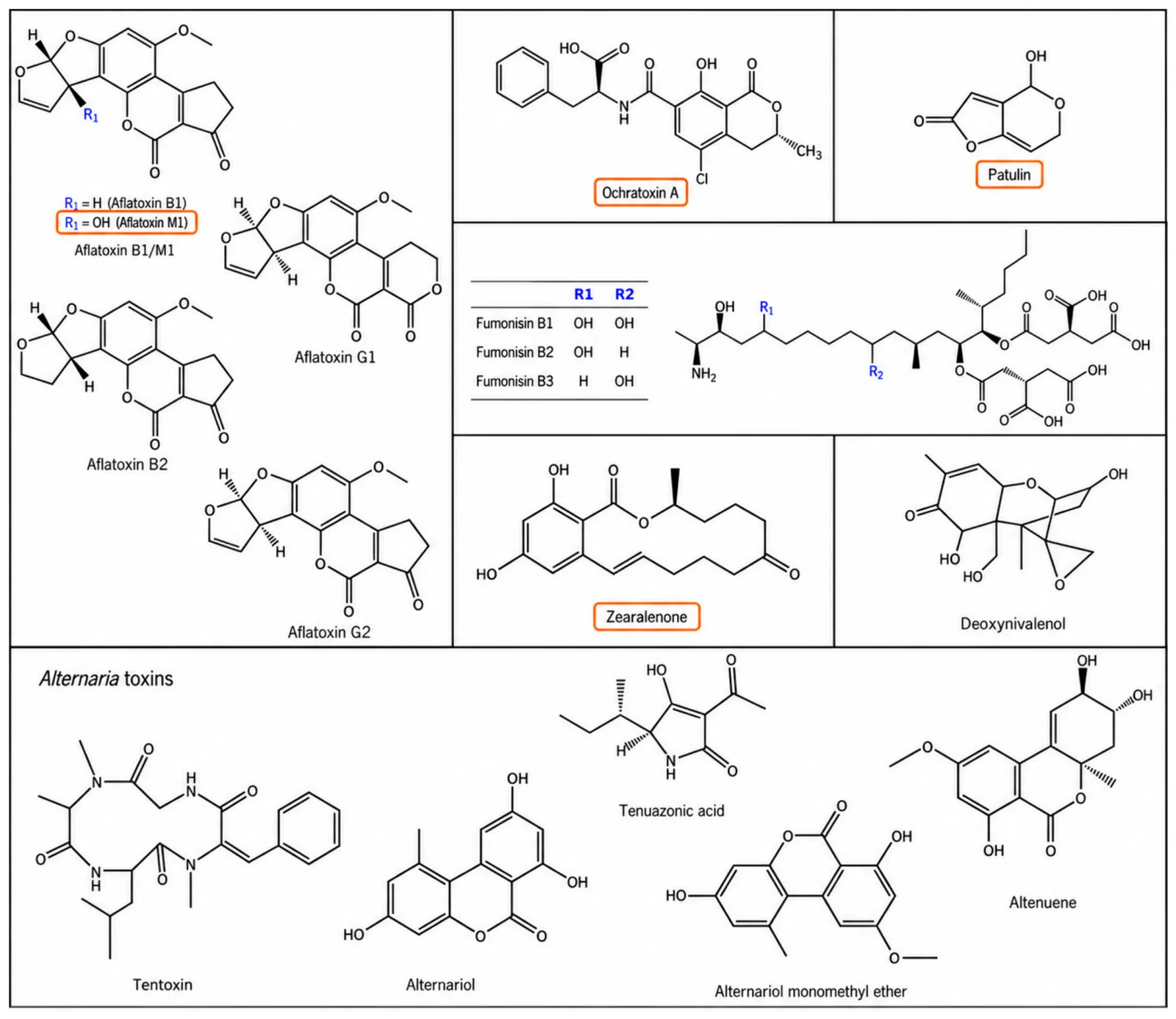
Chemical structures of the main mycotoxins of toxicological relevance (aflatoxins, ochratoxin A, fumonisins, deoxynivalenol, zearalenone, patulin, and *Alternaria* toxins). The mycotoxins highlighted with a rectangle are those that have already been degraded by lipases or, in the case of zearalenone, by zearalenone hydrolases (ZHD) (Section 2.1).

Mycotoxins are natural contaminants of major concern in food and feed due to their high toxicity, stability, and the challenges associated with achieving their safe and efficient removal. It is estimated that more than 300 mycotoxins have already been identified, although only a few are subject to regulation due to their high prevalence and toxicity [1]. Among the most studied are aflatoxins (AFs), ochratoxin A (OTA), fumonisins (FUMs), deoxynivalenol (DON), zearalenone (ZEN), patulin (PAT) and, more recently, *Alternaria* toxins (Figure 1). These mycotoxins may occur individually or co-occur, which increases the risk to human and animal health and requires specific mitigation strategies [2,3,4]. These concerns have also driven the development of multi-mycotoxin analytical approaches capable of simultaneously monitoring several regulated toxins in food matrices, as demonstrated for maize [5].

Mycotoxin mitigation remains challenging, and prevention through good agricultural and storage practices continues to be essential, although it is not always sufficient. Physical methods such as heat, irradiation and adsorption, and chemical approaches, such as ozonation, ammoniation and peroxidation, when allowed, have been applied for their mitigation. However, these methods present important limitations, including incomplete detoxification, loss of nutritional value, organoleptic changes and, in the case of chemical treatments, risks associated with the formation of toxic by-products [6,7,8,9]. Under current European Union legislation, food containing contaminants listed in Annex I of Regulation (EU) 2023/915 may not be deliberately detoxified by chemical treatments [10]. For specific food categories for which dedicated maximum levels are established, sorting or other physical treatments may be applied before the product is placed on the market for the final consumer or used as a food ingredient, provided that the applicable maximum levels are subsequently met [10]. The regulatory framework for feed is different, as physical, chemical, and biological detoxification processes may be permitted under specific conditions and safety requirements [11].

To address the limitations of conventional mitigation approaches, enzymatic degradation has emerged as a sustainable alternative for mycotoxin mitigation, with growing interest in its potential application in different contexts of the agri-food sector. This strategy offers the advantage of eliminating the need for additional nutrient supplementation while preventing the accumulation of microbial biomass [12]. It is considered a promising approach with a lower potential to compromise the nutritional value of food; however, its safety should be evaluated considering transformation products, residual process components, DES-related residues, and matrix effects [13]. A key distinguishing feature is the high specificity of enzymatic processes, which tend to generate fewer secondary residues and increase the probability of obtaining cleaner final products [14]. Several enzyme classes, including oxidoreductases and hydrolases (e.g., lactonase and lipases), have already demonstrated the ability to effectively degrade mycotoxins, leading to the formation of transformation products that may exhibit reduced toxicity when this has been experimentally demonstrated [6]. 

Among these enzymes, lipases are particularly relevant to the scope of this review. Lipases belong to the hydrolase family and are found in microorganisms, plants and animal tissues. These enzymes are highly versatile since they can catalyze a wide range of reactions including hydrolysis, alcoholysis, acidolysis, transesterification and esterification [15]. They have attracted particular interest due to their potential to degrade different mycotoxins, such as ochratoxins, patulin, zearalenone and aflatoxin M1. Nevertheless, method optimization, especially the assessment of solvent effects, remains a crucial step for advancing enzymatic reactions. In this context, the reaction medium becomes particularly important as it can strongly influence enzyme stability, substrate accessibility and overall catalytic performance.

Deep eutectic solvents (DESs) are of particular interest in this context because of their tunable physicochemical properties and their potential use in biocatalytic systems. At the same time, these solvents have been extensively explored as alternative for the green extraction of valuable compounds from agri-food materials. This dual application provides a direct connection between enzymatic mycotoxin mitigation and by-product valorization [16,17].

Within the circular economy framework, agri-food waste and by-products have gained increasing attention because their valorization can reduce waste disposal, improve resource efficiency, and recover valuable compounds for new food, pharmaceutical, cosmetic, and other industrial applications. This approach contributes to making production systems more sustainable and is closely aligned with the United Nations Sustainable Development Goals, particularly SDGs 12 and 13, which concern responsible consumption and production, and climate action, respectively [18,19]. In this context, agri-food by-products are increasingly recognized as strategic resources with significant potential for valorization. However, their possible contamination with mycotoxins presents an additional challenge, hindering their safe and sustainable utilization.

Previous reviews have addressed individual aspects of the topics covered here, including enzymatic mycotoxin degradation, DES-based biocatalysis, lipase engineering, DES-assisted bioactive extraction, and agri-food by-product safety. However, these areas have largely been reviewed separately, with no representative review combining all these aspects. Table 1 summarizes these differences and highlights the integrative perspective of the present work.

Based on this gap, the central question addressed in this review is whether DESs can provide a common framework for the sustainable valorization of agri-food by-products and the development of lipase-based strategies for mycotoxin mitigation. To address this question, the influence of the reaction medium and enzyme engineering strategies, including immobilization and chemical modification, on lipase performance is also critically considered.

**Figure 2:**
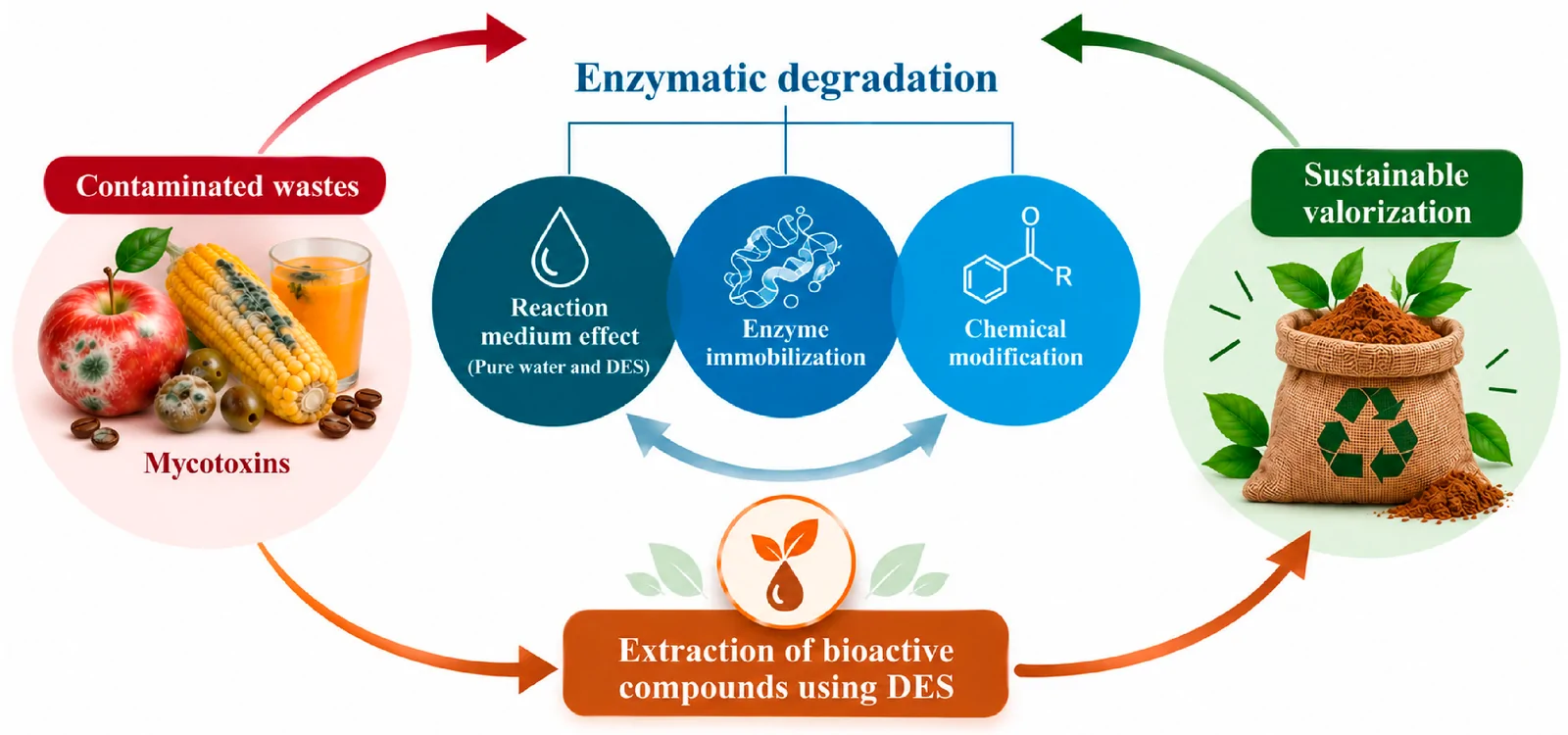
Conceptual overview of the research areas discussed in this review. DES-assisted extraction of bioactive compounds and lipase-mediated mycotoxin degradation are presented as complementary strategies for the valorization of mycotoxin-prone agri-food by-products.

The overall concept discussed in this review is illustrated in Figure 2. The review is organized into four main sections:

(a) The influence of the reaction medium on lipase-mediated mycotoxin degradation, comparing simplified systems and real food matrices; 

(b) The potential of deep eutectic solvents (DESs) as alternative media compatible with lipase activity; 

(c) Strategies to improve lipase performance, including immobilization and chemical modification; 

(d) The application of DESs in the extraction of bioactive compounds from matrices potentially contaminated with mycotoxins.

This article is a narrative review based on representative literature selected to provide a critical overview of the main research areas discussed; it was not conducted as a systematic review.

*Literature search and selection*

This narrative review was based on a literature search performed in Google Scholar and PubMed, complemented by screening of the reference list of the relevant reviews and primary studies selected from the initial search. Searches were conducted using combinations of terms:

- “mycotoxin AND (enzyme OR lipase) AND (degradation OR detoxification)”;
- “(deep eutectic solvent OR DES OR NADES) AND (lipase OR enzyme OR biocatalysis)”;
- “lipase AND (immobilization OR immobilisation OR chemical modification)”;
- “(deep eutectic solvent OR DES OR NADES) AND (agri-food by-product OR waste OR pomace) AND (extraction OR valorization)”, and “mycotoxin AND (agri-food by-product OR waste OR pomace) AND (occurrence OR contamination OR safety)”.

The literature search was performed in January 2026 and was updated in October 2026. Only peer-reviewed articles and reviews published in English were considered; these were first checked based on the title and then on the abstract. As this was a narrative review, no formal systematic screening or PRISMA-based selection procedure was applied.

## 2. Effect of the Reaction Medium on Mycotoxin Degradation

The choice of reaction medium is a critical factor as it directly impacts the effectiveness of lipases in mycotoxin degradation. The reaction environment influences enzyme activity, stability, selectivity, and even conformation, meaning that a single lipase can exhibit markedly different performance under varying conditions. Increasing evidence shows that lipases can hydrolyze amide bonds, as in the case of OTA, and lactone bonds, such as those in PAT, generating less toxic or non-toxic products.

Liu et al. [31] demonstrated over 90% degradation of PAT (5 µg/mL) in phosphate-buffered saline (PBS) (pH 7.5, 40 °C, 48 h) using 0.36 mg/mL of porcine pancreatic lipase (PPL), identifying an ion at m/z 159.0594 as the main product. This finding is consistent with lactone ring opening and supports a hydrolytic mechanism. These results indicate that PPL can hydrolyze PAT under the tested buffered aqueous conditions.

In parallel, a 2022 review emphasized that, while zearalenone hydrolases (ZHDs) have already shown efficacy in real matrices, classical lipases remain mostly evaluated under in vitro conditions, underscoring the importance of the reaction context [32].

**Figure 3:**
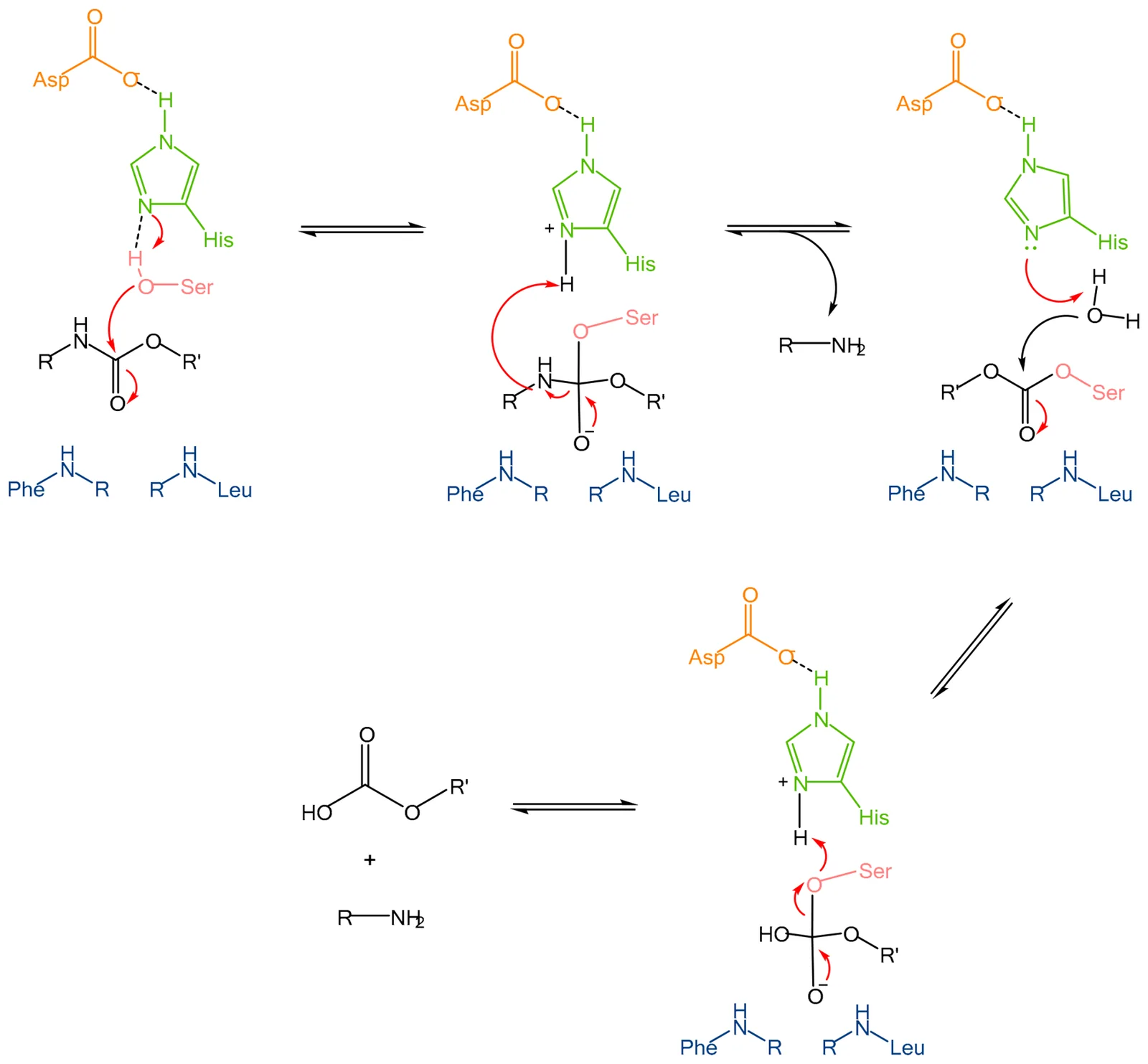
Proposed schematic mechanism of amide-bond hydrolysis by a lipase containing the Ser–His–Asp catalytic triad. Aspartate, histidine, and serine residues are represented in orange, green, and pink, respectively, while phenylalanine and leucine residues are shown in blue. Red curved arrows indicate electron-pair movement during the proposed catalytic mechanism.

A study evaluated 23 commercial hydrolases for OTA degradation and identified a crude lipase from *Aspergillus niger* (Amano A) as the only enzyme capable of effectively hydrolyzing the mycotoxin, with a final specific activity of 2.32 U/mg [33]. Kholif et al. [34] reported 77% degradation of OTA after 18 hours in phosphate-buffered saline, a simplified model medium, using bacterial enzymes. Despite the growing number of studies on enzymatic degradation, direct comparisons of reaction media in lipase-based mitigation remain scarce. Simplified media may suggest high efficiencies, but do not necessarily translate to real food matrices. In the case of amide-bond hydrolysis catalyzed by a lipase containing the Ser–His–Asp catalytic triad, the reaction begins with histidine acting as a base (Figure 3).

It removes the proton from the hydroxyl group of serine, converting serine into a highly reactive nucleophile. The deprotonated serine then attacks the carbonyl carbon of the amide, forming the first tetrahedral intermediate, in which the former carbonyl oxygen carries a negative charge that is stabilized through hydrogen bonding within the oxyanion hole (phenylalanine and leucine). This tetrahedral intermediate quickly collapses, re-forming the carbonyl and breaking the amide C–N bond. The protonated histidine donates its proton to amine, which leaves as R′–NH_2_, and the reaction proceeds through the formation of an acyl–enzyme intermediate, where the acyl group remains covalently attached to the serine residue [35,36,37].

In the next step, a water molecule is activated by histidine, generating a nucleophilic species that attacks the carbon. This produces a second tetrahedral intermediate, again stabilized by the oxyanion hole. Its collapse breaks the bond between the acyl group and serine and restores the serine hydroxyl. The reaction concludes with the release of carboxylic acid and the amine, returning the enzyme to its initial state and allowing the catalytic cycle to begin anew.

Both classical screening studies and recent reviews consistently identify lipases as leading candidates among hydrolases for mycotoxin degradation [33,38].

### 2.1. Degradation in Real Matrices Using Lipases

The application of lipases in real and complex matrices represents an additional challenge since these media contain various constituents capable of modulating or even inhibiting enzymatic activity. Several studies have shown that phenolic compounds, anthocyanins, and other secondary metabolites can directly interact with enzymes, reducing their catalytic efficiency [39,40,41,42]. This helps explain why enzymatic activity measured in model systems is often overestimated when compared with real food matrices. 

More broadly, studies on enzymatic bioremediation emphasize that although the use of enzymes (including lipases) represents a promising ecological alternative, adverse environmental and chemical factors may partially inactivate the enzyme [43]. 

In the case of aflatoxin M1 (AFM1), bacterial lipases and proteases showed high degradation capacity in PBS at 37 °C, reaching 57% (lipase) and 73% (protease) after 18 h. In yogurt, however, degradation ranged from 23% to 66% after 2 days [44].

Similarly, Wang et al. [16] conducted a screening in phosphate buffer (pH 7.4, 37 °C, 24 h) and subsequently tested the enzymes in wine and grape juice (37 °C, 48 h) for OTA degradation. In buffer, carboxypeptidase A (CPA) and a lipase (origin not specified) completely degraded the mycotoxin. In real beverages, the lipase exhibited lower performance, degrading 21% of the mycotoxin in wine and 39% in grape juice after 48 hours, while CPA maintained good performance.

In a study with pear juice contaminated with PAT, a concentration of 0.375 µg/mL resulted in 61% degradation after 24 hours at 40 °C using PPL (20 mg/mL) [45]. Compared with the assays performed in PBS, where PPL achieved 90% PAT degradation after 48 hours using only 0.36 mg/mL of enzyme and a much higher toxin concentration (5 µg/mL) [31], its performance in the real matrix was considerably lower. In pear juice, even with a 50-fold higher enzyme dose and substantially lower PAT levels, degradation dropped to about 61%.

Another example is the application of a specialized zearalenone hydrolase (ZHD), specifically a lactonase rather than a classical lipase, for ZEN degradation. Although ZHDs constitute a distinct enzyme class, they belong to the α/β-hydrolase superfamily and employ a serine-based catalytic mechanism that is mechanistically related to that of lipases. Researchers applied the ZLHY-6 variant during corn oil refining, reducing ZEN from 1257 to 13 µg/kg, which is below the EU limit for refined oils (400 µg/kg). The degradation exceeded 96%, and the hydrolyzed products were removed during the washing step [46]. Importantly, the process did not significantly alter the content of tocopherols or sterols, demonstrating the feasibility of applying this approach in real oil matrices. These findings support the potential application of specialized hydrolases in complex matrices.

Taken together, these studies show that enzymatic performance is strongly influenced by the experimental context and matrix complexity. Direct comparison between studies is limited by differences in enzyme source and dose, initial mycotoxin concentration, reaction time, experimental conditions, and analytical approaches. These methodological differences should therefore be considered when interpreting apparent variations in degradation efficiency.

### 2.2. DESs as Alternative Reaction Media

Beyond buffer solutions, DESs have emerged as promising alternative media in biocatalysis. Depending on their composition, they may offer advantages such as simple preparation, low volatility, biodegradability, and biocompatibility; however, these properties cannot be generalized to all DES formulations. These liquids are formed by combining a hydrogen bond donor (HBD) and acceptor (HBA), generating mixtures with melting points lower than those of the individual components. Owing to their unique properties, DESs have also proven effective in a wide range of enzymatic processes [21].

In recent years, significant advances have been made in the use of DESs in lipase-catalyzed reactions, with several studies showing that these enzymes can maintain, or even enhance, their performance in suitable solvents. In this context, DESs have been increasingly explored as reaction media for different reactions with free or immobilized biocatalysts [22,23].

**Figure 4:**
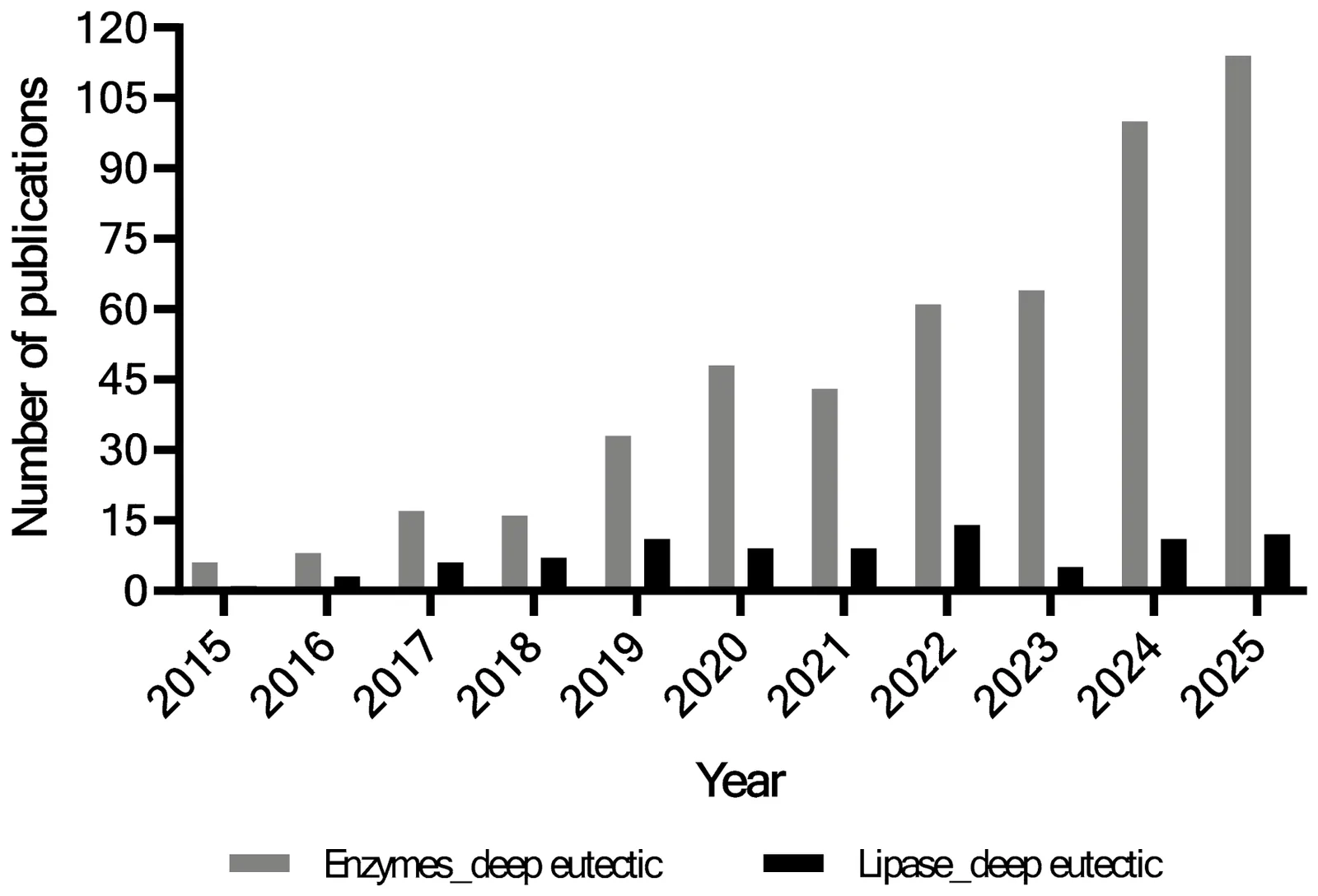
Indicative evolution of publication counts related to the searches “enzymes_deep eutectic” and “lipase_deep eutectic” on PubMed and Google Scholar between 2015 and 2025. The values are presented to illustrate general research interest over time and should not be interpreted as a formal bibliometric analysis or as evidence of scientific effectiveness.

To provide an indicative overview of the evolution of research interest in DES-related enzymatic processes, searches were performed in PubMed and Google Scholar using the terms “enzymes_deep eutectic” and “lipase_deep eutectic” (Figure 4) for the period of 2015–2025. This time frame was selected because DESs began to appear more consistently in the literature from 2015 onwards, accompanied by a marked increase in their application in biocatalysis. For each year, the total number of articles retrieved from both platforms was recorded, providing a clear visualization of the evolution of scientific interest over the past decade. The results reveal a steady growth, reflecting the expanding application of DESs in enzymatic systems, particularly in lipase-catalyzed reactions. Overall, the observed trend illustrates the increasing research interest in DES–enzyme and DES–lipase systems over the last decade.

To the best of our knowledge, the use of lipases in deep eutectic solvents for the enzymatic degradation of mycotoxins remains virtually unexplored. Studies combining DESs with mycotoxins have focused almost exclusively on extraction and analytical applications, while DES–lipase systems reported in the literature concern other types of biocatalytic reactions [47,48,49]. Even so, research on related enzymatic processes in DESs offers valuable insights that help predict the potential of this strategy. Noro et al. [50] tested three lipases (*Aspergillus oryzae*, *Candida rugosa*, and porcine pancreas) in three DESs (choline chloride (ChCl):urea, ChCl:glycerol (Gly), and TBAB:imidazole) for glucose ester synthesis. All retained hydrolytic activity comparable to aqueous media, while transesterification efficiency varied with the lipase–DES system. Substrate polarity also influenced results, with hydrophilic substrates performing better in ChCl:urea. Overall, DESs support efficient biocatalysis without loss of enzyme activity. Freitas et al. showed that certain DESs not only preserve lipase activity but significantly enhance it, especially when the DES modulates the enzyme–water interaction. The study demonstrates that there is an optimal water activity window in which the lipase reaches its maximum performance, a behavior that is far more favorable than in conventional organic solvents [51]. Another study showed that *Candida antarctica* lipase B (CALB) can be activated by hydrogen bond interactions in ChCl:Gly, which increased its hydrolytic activity toward nonpolar model substrates such as *p*-nitrophenyl hexanoate [52].

It is worth noting that not all DESs are equally favorable to lipases. Liu et al. [53] demonstrated that hydrophilic DESs, such as ChCl:ethylene glycol or ChCl:levulinic acid, are generally well tolerated and may even enhance enzymatic activity. In contrast, a hydrophobic DES (thymol:2,6-dimethoxyphenol) proved to be strongly inhibitory. This is likely because hydrophilic DESs help preserve protein structure and hydration, whereas hydrophobic DESs displace essential water or disrupt hydrophobic regions, leading to enzyme destabilization.

The tunable structure of DESs allows the optimization of reaction selectivity, yield, and rate, making these solvents particularly promising for sustainable and efficient biocatalytic processes [54]. In addition, the tunable solvent properties of DESs may influence the solubility of poorly water-soluble mycotoxins, such as zearalenone or aflatoxins; however, whether this translates into enhanced enzymatic degradation remains to be established. Direct evidence of lipase-mediated mycotoxin degradation in DESs remains very limited. Santos et al. [55] demonstrated OTA degradation by porcine pancreatic lipase in glycerol-based DESs containing a high water content, both in model systems and directly in grape pomace extracts. Most other DES–lipase studies, however, concern esterification, transesterification, hydrolysis of model substrates, polymerization, or related biocatalytic applications. Therefore, although these studies support the feasibility of DES–lipase systems, their application to mycotoxin degradation requires further investigation, particularly in real and complex food matrices.

Overall, the effect of DESs on lipase activity depends strongly on the specific lipase–DES–substrate system. Water activity appears to be particularly important, as an optimal hydration level can favor enzyme performance, while substrate polarity and DES composition influence substrate–solvent and enzyme–solvent interactions. Hydrogen-bonding interactions may also contribute to enzyme activation in some systems, whereas other DES compositions, particularly hydrophobic systems, can be inhibitory. These observations indicate that DESs cannot be considered universally beneficial or detrimental to lipase activity, and their effect depends on solvent composition, water content, substrate properties, and the specific enzyme employed.

## 3. Immobilization of Lipases

Enzyme immobilization has become a central strategy for enhancing the efficiency, stability and sustainability of biocatalytic systems, effectively overcoming the inherent limitations of free enzymes, such as low operational stability, short lifetime and difficulties in recovery and reuse [56]. The classical techniques (adsorption, entrapment, encapsulation, ionic bonding, covalent attachment and cross-linking) have been extensively studied and allow the fine-tuning of the enzyme microenvironment, varying the binding strength and susceptibility to leaching. The choice of support material has likewise proved crucial for maximizing mechanical stability, enzyme loading capacity and alignment with green chemistry principles. Biopolymers and agro-industrial residues rich in cellulose, hemicellulose and lignin have emerged as sustainable, low-cost and versatile supports [57]. Their chemical modification can further enhance enzyme affinity, reduce leaching and improve the thermal and chemical stability of the resulting biocatalysts.

Among the various classes of enzymes, lipases stand out due to their broad applicability in both hydrolytic and synthetic reactions, and they have therefore been widely immobilized on polymeric, inorganic, magnetic and nanostructured supports [58,59].

**Figure 5:**
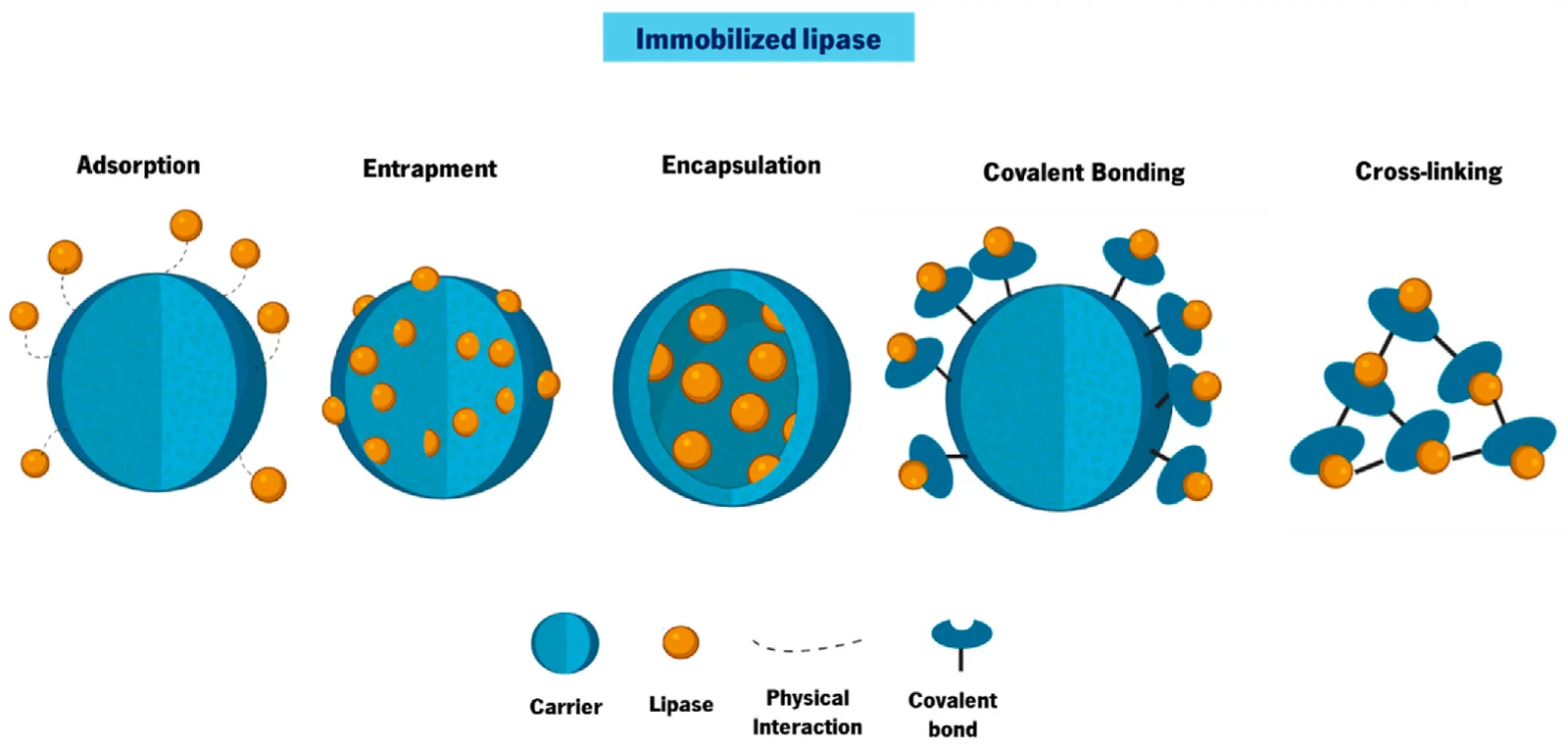
Schematic representation of enzyme immobilization strategies, including adsorption, entrapment, encapsulation, covalent bonding, and enzyme cross-linking.

For lipases in particular, immobilization strategies such as adsorption, entrapment/encapsulation, covalent bonding and the formation of cross-linked enzyme aggregates (CLEAs) (Figure 5) have demonstrated significant advantages, including enhanced thermal and operational stability, mitigation of inhibitory effects and efficient reuse over multiple catalytic cycles [60,61]. These findings indicate that immobilization not only facilitates catalyst recovery but also broadens the operational window and improves performance in complex reaction media.

In recent years, increasing attention has been given to the application of immobilized lipases in sustainable bioprocesses, particularly within the food industry, where they support clean-label processing, waste reduction and the replacement of harsh chemical reagents. A recent review highlighted clear advantages of lipase immobilization in the production of structured lipids, the modification of edible oils, dairy processing and additive development, combining catalytic efficiency with food safety requirements [61]. This trend demonstrates that, beyond academic interest, lipase immobilization is becoming a key enabling technology in industrial sectors that prioritize green, efficient and economically viable solutions.

Recent examples illustrate the diversity of supports explored. Lipases immobilized on silica modified with carbon nanotubes showed higher activity and longer operational life compared to free form [62]. Nanomaterials, such as graphene, polymeric fibers and metal–organic frameworks (MOFs), demonstrated enhanced stability, higher catalytic activity and strong reusability [63]. Poly(ether sulfone) membranes functionalized with amino groups preserved *Mucor miehei* lipase activity after several cycles [64]. Agro-industrial residues, such as apple pomace and chokeberry pomace, also showed potential as sustainable lignocellulosic supports, improving the stability and synthetic activity of lipases while reducing the environmental impact associated with waste [65].

Despite advances in nanostructured supports and green approaches using agro-industrial residues, studies combining immobilization with DESs remain limited. Table 2 provides a summary of the main lipase immobilization methods applied in DES-based systems. Nanodiamonds have been shown to be highly effective support for lipases. *Rhizopus niveus* lipase immobilized on these nanomaterials exhibited an activity approximately 3.5 times higher than the free enzyme, showed enhanced thermal and pH stability, and retained 76% of its initial activity over successive cycles when used in DESs [66].

Guajardo et al. reported the immobilization of *Candida antarctica* lipase B (CALB) in Cross-Linked Enzyme Aggregates (CLEAs) subsequently encapsulated in LentiKats® (PVA hydrogel) and applied in DES–buffer mixtures. The derivative showed high operational stability, achieving complete conversions in the esterification of glycerol, with reuse over six cycles without significant loss of activity [67]. The immobilization of *Pseudomonas stutzeri* lipase using CLEA technology was later explored, aiming to enhance its activity and stability in DESs. While the free enzyme was nearly inactive in ChCl:Gly media, enzyme-CLEA derivatives exhibited esterification activity and reusability across multiple cycles, confirming immobilization as an essential requirement for functioning in such systems [68].

Bellou et al. [69] developed 3D-printed enzymatic microreactors in which CALB was covalently immobilized on polylactic acid (PLA) supports modified with polyethyleneimine (PEI). In betaine:glycerol, the enzyme retained 95% activity and remained stable for 30 days at 60°C. Under continuous flow, the system reached 23-fold higher productivity than batch processing in the transesterification of ethyl ferulate with glycerol, highlighting the potential of DES-PLA-PEI systems for sustainable biocatalysis.

Buzatu et al. [70] evaluated 16 DESs as media for lipase-catalyzed reactions, including sugar ester synthesis. Both free and immobilized lipases retained high activity and thermal stability at 70 °C. The authors tested lipases entrapped in silica-based sol–gel matrices, and the immobilized systems exhibited higher conversions and significantly improved thermal stability, retaining approximately 78–90% of their initial activity after 72 hours. The study demonstrates that combining DESs with immobilized lipases reinforces operational stability and enables sustainable syntheses in biocatalysis.

Similarly, the incorporation of PPL into DES-based eutectogels demonstrated that entrapment within polymeric matrices can greatly enhance the enzyme’s stability and operational robustness [71]. These eutectogels act as hybrid systems in which the DES functions both as a solvent and as a structural component of the gel network.

More recently, Santos et al. [72] investigated the immobilization of porcine pancreatic lipase onto polyamide 6 (PA6)-based microparticles for OTA degradation in grape pomace extract obtained using a glycerol:glucose DES (1:1) with 90% pure water. The PA6 support achieved approximately 80% OTA degradation after 24 h at 44 °C compared with approximately 55% for the free enzyme, demonstrating the beneficial effect of immobilization in complex matrices. Reusability was evaluated over seven consecutive cycles, although a progressive decrease in activity was observed, particularly in grape pomace extract. These results provide direct evidence of OTA degradation by immobilized lipases in DES-derived extracts, highlighting the potential of this approach for mycotoxin mitigation.

Overall, lipase immobilization offers important advantages, including improved operational stability, greater tolerance to complex reaction media, and the possibility of enzyme recovery and reuse. Nevertheless, further studies are required to assess the performance and long-term stability of immobilized lipases in different DES-containing systems, particularly for mycotoxin degradation in real matrices.

**Table 2:** Overview of the main lipase immobilization methods applied in deep eutectic solvents (DESs), including DES composition and molar ratio, reaction conditions, catalytic performance, and reusability, where reported.

Immobilization	Support	Enzyme	Reaction Type/Substrate	DES (Molar Ratio)	Reaction Conditions	Key Findings	Ref.
Adsorption	Nanodiamonds	*Rhizopus niveus* lipase	Hydrolysis of p-nitrophenyl palmitate	Choline chloride:sucrose:water (4:1:4)	5 mg/mL of enzyme immobilized on nanodiamonds (0.5% of total volume), 40 °C, pH 7.0, 1 h	6 reuse cycles tested; 76% activity retained after the first reuse cycle and ~20% after cycles 3–6. Approximately 16% higher activity than free lipase	[66]
	Polyamide 6 (PA6) microparticles	Porcine pancreatic lipase	Hydrolysis of OTA	Grape pomace extract containing glucose:glycerol DES (1:1), diluted to 90% water	24 U/mg enzyme; 1 µg/mL OTA; 44 °C; 4–24 h	7 cycles; > 50% activity retained in water after 7 cycles. ~80% OTA degradation vs. ~55% with free PPL in grape pomace extract (24 h)	[72]
Covalent bonding	PLA–PEI (3D printed)	*Candida antarctica* lipase B	Hydrolysis of p-nitrophenyl butyrate; transesterification of ethyl ferulate	Betaine:glycerol (1:3)	CALB immobilization solution (0.044 mg/mL); hydrolysis: 10% (*v*/*v*) DES, 40 °C, pH 7.5; transesterification: 100% DES, 60 °C, 4 h	100 cycles with ~100% activity retained (hydrolysis); 1.2-fold higher productivity than free CALB (transesterification)	[69]
Cross-linking (CLEAs)	______	*Pseudomonas stutzeri* lipase	Esterification of benzoic acid with glycerol (α-MBG synthesis)	Choline chloride:glycerol (1:2)	300 mg biocatalyst/10 mL DES; phosphate buffer (100 mM, pH 7.0); 60 °C; 24 h per batch	5 batches; conversion decreased from ~10% to ~3%. Free lipase was inactive in DES, whereas TL-CLEA retained catalytic activity	[68]
Encapsulation	LentiKats® (PVA hydrogel)	*Candida antarctica* lipase B (as CLEAs)	Esterification of benzoic acid with glycerol (α-MBG synthesis)	Choline chloride:glycerol (1:2)	150 mg biocatalyst/mL; 100 mM benzoic acid; 10% (*v*/*v*) phosphate buffer (100 mM, pH 7.0); 60 °C; 24 h per reuse cycle	6 reuse cycles; ~65% conversion, 16 percentage points higher than CALB-CLEA. No free-enzyme comparison	[67]
Entrapment	Sol–gel matrix	*Candida antarctica* lipase B *Pseudomonas stutzeri* lipase	Esterification of D-arabitol with lauric acid	Choline chloride:D-arabitol (1:1)	300 U immobilized CALB; 1.6 mmol lauric acid; 70 °C, 600 rpm, 67 h	23% lauric acid conversion; no reuse cycles or direct free-enzyme comparison reported for this reaction	[70]
	Eutectogels	Porcine pancreatic lipase	Hydrolysis of p-nitrophenyl acetate and p-nitrophenyl palmitate	Tetramethylammonium chloride:glycerol (1:2)	1.75 mM enzyme in eutectogel (175 µM effective assay concentration); TRIS buffer (100 mM, pH 8.2); 37 °C	No reuse cycles reported; >60% activity remained after 35 days of storage. Free lipase showed faster hydrolysis than immobilized lipase	[71]

## 4. Chemical Modification of Lipases

Chemical modification of enzymes has long been an important strategy for enhancing their stability and catalytic performance under demanding operational conditions. Lipases exhibit remarkable versatility, catalyzing a wide range of reactions with high industrial relevance. As a result, optimizing their properties has become a major focus in biotechnology [24].

**Figure 6:**
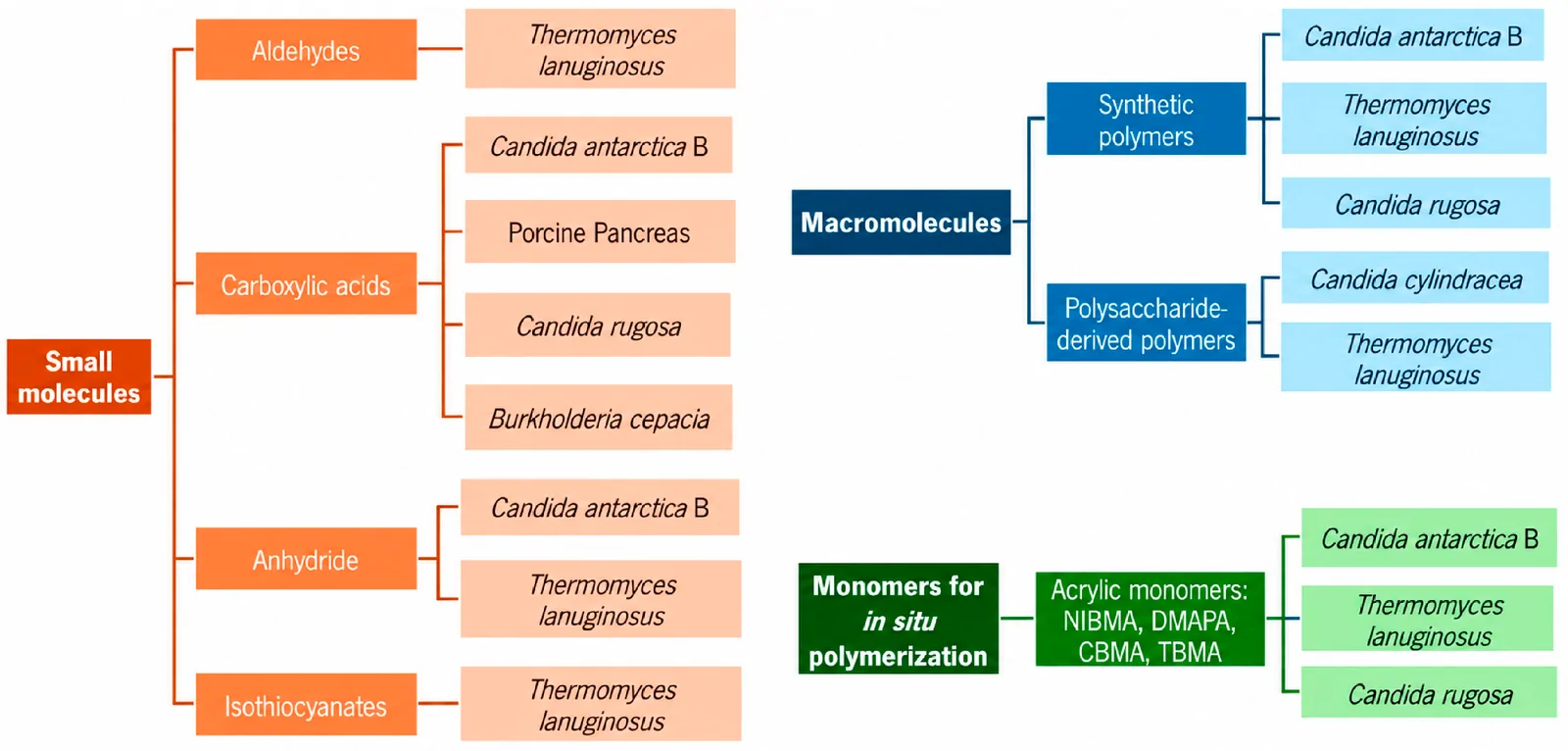
Main classes of molecules (small molecules, macromolecules, and monomers for in situ polymerization) used for lipase chemical modification and the corresponding lipase sources reported in the literature.

One of the most common approaches is the modification of surface residues using small molecules. This strategy is mainly employed to improve catalytic properties but can also reinforce characteristics, such as thermostability, tolerance to organic solvents, and enantioselectivity [73,74]. The modification typically involves reacting functionalized small molecules that bear aldehyde, carboxylic acid, anhydride or isothiocyanate groups, with lysine residues exposed on the enzyme surface, forming covalent bonds (Figure 6).

Modification of *Candida antarctica* lipase B (CALB) using various ionic liquids containing carboxyl groups has been shown to enhance hydrolytic activity, improve thermal stability, and increase tolerance to organic solvents [75]. Similar trends have been observed for lipases from porcine pancreas [76], *Burkholderia cepacia* [77], and *Candida rugosa* [78], confirming that this type of grafting is particularly effective in making lipases more robust and versatile.

Other small reagents, such as benzoic anhydride, have also been used to modify CALB and *Thermomyces lanuginosus* lipase (TLL), yielding substantial improvements in thermal and long-term stability [79]. Additional work modifying TLL with different aldehydes (naphtaldehyde, butyraldehyde, hexyl aldehyde and dodecyl aldehyde) and naphthyl isothiocyanate showed that long-chain aldehydes led to improved thermostability and, notably, significantly increased both hydrolytic and synthetic activity [74]. In line with these results, the chemically modified TLL was also evaluated for its esterification performance using linear alcohols ranging from C1 to C20 [80]. The enzyme grafted with four dodecyl chains showed markedly superior catalytic activity compared with the native form, with performance increasing with alcohol chain length. This confirms that the modification enhances both thermostability and substrate scope in synthesis reactions.

Beyond small molecules, covalent coupling of polymers represents one of the most widely used strategies for modifying lipases (Figure 6). Among the various polymers investigated, PEGylation clearly stands out as the most common. This process involves covalently attaching polyethylene glycol (PEG) chains to small molecules or macromolecules, and has gained prominence due to the advantageous properties of PEG, including low toxicity, enhanced stability, and improved therapeutic performance [81].

PEGylation with 5 kDa PEG enhanced the hydrolytic activity of CALB and preserved that of TLL. Regarding the ability to catalyze polymerization reactions, PEGylation resulted in a pronounced improvement for TLL, whereas CALB showed a performance comparable to that of the native enzyme in the synthesis of poly(ethylene glutarate) [82]. Alternative polymers such as Pluronic F-127 have likewise been shown to enhance activity in transesterification reactions and enable efficient reuse of the biocatalyst thanks to temperature-induced precipitation [83,84,85]. Other macromolecular strategies, including the use of polysaccharide-derived polymers [86,87,88], mainly confer improvements in thermal stability and solvent tolerance. Additionally, modification of *Bacillus subtilis* lipase with poly(4-acryloylmorpholine) has resulted in increased transesterification activity in ionic liquids [89].

More recent strategies include in situ polymerization (Figure 6) on the enzyme surface using acrylic monomers such as N-isobutyl methacrylate (NIBMA), dimethylaminopropyl acrylate (DMAPA), carboxybutyl methacrylate (CBMA), and tert-butyl methacrylate (TBMA), yielding hybrid biocatalysts with enhanced activity, greater thermal stability, and improved substrate affinity [90,91]. Although acrylic monomers are technically small molecules in terms of size, they are not considered small-molecule modifiers. 

Ionic liquids have been widely used as modifying molecules because they can be easily functionalized with reactive groups capable of forming covalent bonds with enzymes. In contrast, DESs lack a defined molecular structure and do not possess reactive functional groups. Although promising results have been reported in other applications, chemically modified lipases have not yet been investigated in DES-containing systems. Their potential relevance to mycotoxin degradation therefore lies primarily in their demonstrated ability to improve enzyme stability, solvent tolerance, and substrate affinity.

## 5. Mycotoxin Contamination and Valorization of Agri-Food By-Products

Mycotoxin contamination in agri-food products and by-products poses a significant risk to food safety but also a major challenge for the valorization of these materials within the framework of the circular economy. Residues such as grape pomace, apple pomace, tomato by-products and other derivatives from the agri-food industry are rich in bioactive compounds of high interest, recognized for their antioxidant activity and health benefits. However, their susceptibility to fungal contamination raises additional concerns. In this context, the application of innovative strategies such as the use of DESs for the sustainable extraction of bioactive compounds emerges as a promising approach, capable of combining safety, sustainability and efficient valorization of these by-products.

**Figure 7:**
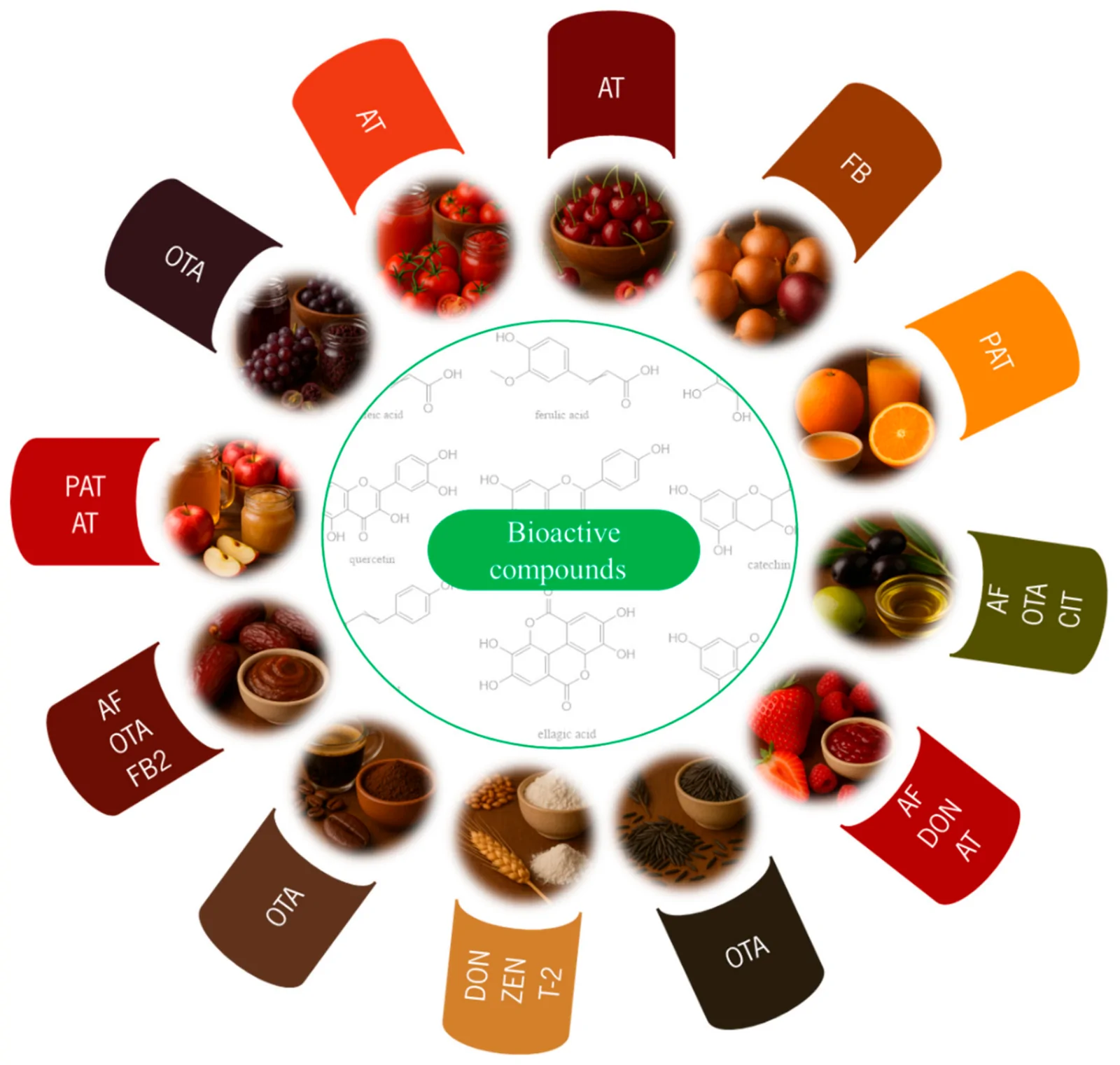
Representation of the different food matrices and the most frequently detected mycotoxins, highlighting the added-value bioactive compounds. The mycotoxins represented include aflatoxins (AF), ochratoxin A (OTA), patulin (PAT), fumonisin B (FB), deoxynivalenol (DON), zearalenone (ZEN), T-2 toxin (T-2), citrinin (CIT) and *Alternaria* toxins (AT).

Figure 7 illustrates the duality between the potential for bioactive compound recovery and the occurrence of mycotoxins in agri-food by-products, highlighting the need for safe and sustainable valorization strategies.

### 5.1. Winery By-Products and Wastes

Grapes (*Vitis* spp.) are among the most valued fruits worldwide, with an annual production exceeding 73 million tons. Grape pomace, the main by-product of winemaking, represents about 20–25% of the total weight of processed grapes and consists of skins, seeds, stems and small portions of pulp [92]. This matrix is particularly rich in structural fibers, proteins and bioactive compounds with diverse activities that can be exploited across multiple sectors. In the food industry, it serves as a source of dietary fibers, natural colorants and texturizing agents. In the pharmaceutical field, it is valued for its antioxidant potential and nutrient supply, while in cosmetics, it is applied for its antimicrobial effects [93]. Despite their high nutritional and functional value, grapes and their derivatives can be contaminated with mycotoxins, particularly OTA, which are mainly produced by *Aspergillus* fungi [94]. The grape skin is particularly susceptible to mycotoxin contamination, as it is directly exposed to environmental factors and represents the first barrier against fungal colonization [95]. This potential occurrence reinforces the importance of systematic monitoring of extracts, especially when intended for food consumption or applications involving direct human contact.

#### 5.1.1. Grape Pomace

In southern Italy, OTA was detected in 100% of samples, with concentrations ranging from 34.2 to 456.8 µg/kg in grape pomace, exceeding the recommended limits for poultry and swine feeds in about 70% of cases [96,97]. In contrast, in Portugal, OTA appeared in 12 of 13 pomace samples but at much lower levels (up to 0.4 µg/kg), with slightly higher values in a more humid subregion [98]. Although considered a limited concern, the authors emphasize the need for regular monitoring, as regional differences reflect the influence of environmental conditions and winemaking practices. In this context, it becomes crucial to adopt strategies that combine safety and efficiency in the valorization of these by-products.

A clear progression can be seen in the use of DESs for grape by-product valorization, with gains in extraction efficiency and, in some cases, in bioactivity and safety. In 2019, Panić et al. [99] scaled up anthocyanin extraction using ChCl:citric acid (2:1, 30% (*v*/*v*) H_2_O), achieving 1.77 mg/g dw with good stability and solvent recyclability. In 2021, Punzo et al. [100] reported high antioxidant activity and 26.02 ± 1.16 mg GAE/mL total polyphenols using betaine:citric acid (40% (*v*/*v*) H_2_O), noting the absence of cytotoxicity, although mycotoxins were not assessed. Vorobyova et al. [101] applied betaine:lactic acid (1:2, 5% (*v*/*v*) H_2_O) for phenolic extraction and subsequent silver nanoparticle synthesis, obtaining extracts rich in hydroxycinnamic acids (23%) and anthocyanins (19%), including gallic acid, caffeic acid, and catechin. Li et al. [102] identified ChCl:lactic acid (1:2, 25% (*v*/*v*) H_2_O) as the most efficient system, reporting eight anthocyanins (delphinidin, peonidin, and petunidin, among others) and high antioxidant activity.

#### 5.1.2. Grape Skin

Two different studies analyzed the role of grape skin in mycotoxin contamination, although with distinct approaches. Gonçalves et al. [95] found no natural contamination but showed high adsorption capacity under simulated conditions, with removal rates of 88-100% for OTA, AFB1, FB2, and ZEN, confirming the skin as a chemical sponge for environmental mycotoxins. In contrast, another study focusing on natural OTA occurrence, mainly produced by *Aspergillus carbonarius*, reported that skins and seeds retain 50-80% of the toxin in crushed grapes [103]. The risk increases as grapes ripen due to softening of the skin and greater susceptibility to damage, especially under warm, humid conditions. Collectively, these findings identify grape skin as a critical point for mycotoxin adsorption and accumulation, warranting targeted monitoring. 

Phenolic extraction from grape skin using DESs has been evaluated in two studies aimed at green, high-efficiency anthocyanin recovery. The first study used citric acid:maltose (4:1, 24% (*v*/*v*) H_2_O), achieving a yield of 63.36 mg/g total anthocyanins, about double the yield of conventional methods, and showing strong correlation with antioxidant activity [104]. In the second study, ChCl:oxalic acid (1:1, 25% (*v*/*v*) H_2_O) extracted up to five times more anthocyanins than water and twice as much as 70% methanol, with its acidity and water content driving selectivity toward polar anthocyanins [105]. However, based on the information available, no toxicity testing or mycotoxin screening of the final extracts was performed.

#### 5.1.3. Wine Lees

Wine lees are solid residues from fermentation, composed of yeast cells, skin fragments, and other sediments. Rich in anthocyanins and other phenolics, they represent a promising source of high-value bioactive compounds [106]. However, the presence of mycotoxins in wine, particularly OTA, is well documented, and their removal during winemaking largely occurs through transfer to the solid fraction, including the lees [107,108].

Several studies have shown that, during winemaking, OTA present in grapes and must is largely removed from wine through adsorption onto lees, reducing the mycotoxin in the final product but concentrating it in these residues [108,109]. Winemaking trials indicate that more than 80% of OTA can be removed during the process, mainly through binding to solids followed by racking/clarification [110]. The main mechanism is adsorption onto the yeast cell wall, which explains why small amounts of lees can reduce OTA by up to 70% within just 7 days, with measurable reductions already occurring within the first few hours [111]. Studies in microvinifications and naturally contaminated wines confirm this effect, with about 50% of OTA retained after alcoholic fermentation, 18% in sediments from natural decantation, and 3% in lees from malolactic fermentation [112]. Thus, the reduction in OTA in the final wine results mainly from the physical removal of these solids, but lees become a critical reservoir of mycotoxins that must be considered in their valorization and subsequent management. Collectively, these findings indicate that grape skin can act as an important site of mycotoxin adsorption and retention, particularly for OTA, thereby contributing to the presence of these contaminants in grape-derived by-products.

Bosiljkov et al. [113] evaluated the use of DESs combined with ultrasound-assisted extraction as a green alternative for extracting anthocyanins from wine lees. Among the seven combinations tested, ChCl:malic acid (1:1, 35.4% (*w*/*w*) H_2_O) was the most promising, and the addition of water proved crucial for reducing viscosity and improving mass transfer. No references to toxicity testing of the extracts were identified, with the study focusing primarily on extraction efficiency and industrial scale-up potential.

### 5.2. Other Agro-Food Wastes and By-Products

#### 5.2.1. Mycotoxin Contamination

In addition to grape-derived products, several other agro-industrial by-products have shown similar contamination potential. Agro-food residues with high water contents, such as apple pomace, are particularly susceptible to microbial colonization and spoilage due to their high moisture and biodegradable organic load [114]. Other fruit residues—including tomato, cherry, strawberry, and date—as well as plant-based by-products such as onion peels, citrus peels, and olive leaves, have also been associated with the presence of multiple mycotoxins. Mycotoxin contamination in agro-food by-products is mainly linked to compounds produced by *Penicillium*, *Aspergillus*, *Fusarium*, and *Alternaria* species.

PAT, produced mainly by *Penicillium expansum*, is the most extensively studied mycotoxin in apples and apple residues, frequently occurring above European limits [115,116,117,118,119]. International reports reveal considerable variability in contamination levels across different regions and products. In China, 16% of 95 analyzed products exceeded 50 µg/kg PAT, and in infant foods, 36.7% surpassed 10 µg/kg, with a maximum value of 94.7 µg/kg in juice concentrate [115]. In another study, the mycotoxin was detected in 97-100% of concentrates but rarely above the European limit [116]. A more severe situation was reported in the United States, where up to 11.3% of samples exceeded regulatory limits, reaching 2700 µg/L [118]. Levels above regulatory thresholds were also found in 10.4% of Argentine samples (maximum: 1962 µg/kg) [117] and in 34.8% of Italian juices (maximum: 55.41 µg/kg), with the highest concentrations observed in beverages with higher fruit contents [119]. In pomegranate, PAT has also been reported in studies from Egypt, but not naturally and occurs alongside *Alternaria* toxins, but only after inoculation with toxin-producing fungi previously isolated from the fruit [120,121]. Studies show that although some cases of higher contamination have been found in processed orange-based products, fresh oranges and their derivatives generally exhibit low patulin levels [122].

Aflatoxins, mainly AFB1 [123,124,125], occur in olive oil and dates, where they coexist with OTA and enniatins [126,127,128,129], and in commercial cinnamon [130,131]. Enniatins are cyclic depsipeptide mycotoxins mainly produced by *Fusarium* species and are frequently found as co-contaminants. Santos et al. [132] analyzed 84 leaf samples from medicinal and aromatic olive plants commercialized in Spain and reported, for the first time, the simultaneous contamination of olive leaves with multiple mycotoxins. In two samples, aflatoxins (78 and 58 µg/kg), ZEN (9 and 43 µg/kg), T-2 toxin (4 and 3 µg/kg), and DON (150 and 127 µg/kg) were detected. In one of the samples, OTA (1 µg/kg) and citrinin (15 µg/kg) were also identified. 

LC-MS² analyses have confirmed the frequent occurrence of multiple mycotoxins, including AFB1, OTA, fumonisins, trichothecenes, and enniatins, in ready-to-consume coffee products such as coffee beans, ground coffee, capsules, and beverages [133,134,135,136,137]. 

OTA occurs across a wide range of matrices, including olive oil [125], dates [127,128] (with the latter constituting the first report of the natural co-occurrence of OTA and FB2 in dates), cinnamon [138], and Chinese wild rice [139].

In apples, *Alternaria* mycotoxins represent the second most relevant group after PAT [140,141,142]. Yinping Li et al. reported the co-occurrence of PAT and Alternaria toxins in 73% of apples and in 4.7% of derived products [140], confirming the persistence of these contaminants from fresh fruit to the final product [143]. Multiple mycotoxins were detected in infant foods, with the combination of AOH and AME exceeding toxicological reference values [141]. AOH was consistently identified in 67% of apple products analyzed, with an average value of 1.5 µg/kg [142].

In tomatoes and derived products, they are frequently detected at high contamination rates, with TeA being the predominant mycotoxin [144,145,146,147,148]. In China, 58.8% of tomato products tested positive, mainly for TeA, AOH, AME, and TEN [144]. Contamination rates were highest in tomato powder and canned tomato (100%), followed by sauces (89.6%), ketchup (82.1%), juice (46.7%), and dried tomato (20%). TeA was most prevalent (56.7%, up to 7.99 µg/kg), often co-occurring with AOH and AME. Fusarium toxins such as enniatins (A, A1, B, and B1) and beauvericin were also detected but at lower incidences (up to 19.6%). Another study in the same country confirmed the importance of TeA, which was detected in 100% of ketchup samples (10.2–1787 µg/kg) and tomato juice samples (7.4–278 µg/kg) [145]. In Brazil, AOH was found in ketchup at concentrations of up to 8.45 µg/g [146]. In Italy, dried tomatoes showed very high concentrations of TeA (up to 81.59 µg/kg), often in co-occurrence with AME and TEN [147]. In Belgium, AOH, AME and TeA were detected, and the mean dietary exposure to TeA reached 4230 ng/kg body weight per day, a value exceeding the toxicological concern threshold set by EFSA (1500 ng/kg bw/day) [148]. In cherries, the first mycotoxins were identified in the fruit [149]. TeA, AOH, and AME have also been detected in strawberries and raspberries [148]. In strawberries, AOH was detected in 42% of samples stored at 22 °C (26–752 ng/g) and in 37% of those kept at 6 °C, while AME was more common under prolonged refrigeration (11–137 ng/g) [150]. Aflatoxins were also detected in more than 70% of the samples from both fruits, but always below 4 µg/kg [151]. DON, however, was found exclusively in raspberries, exceeding 750 µg/kg in 17% of cases [152]. In addition, strawberries showed a higher incidence of P. expansum, whereas raspberries revealed greater Penicillium diversity, suggesting a higher risk associated with the latter fruit [153].

*Fusarium* mycotoxins are particularly relevant in onions, cereals, and tomatoes. Market analyses confirm the presence of fumonisins in commercial onions, in addition to beauvericin and moniliformin [154,155]. In wheat, DON is consistently the dominant mycotoxin, often found co-occurring with ZEN, T-2, and enniatins [156,157,158]. In raspberries, DON reaches particularly high levels [152].

**Figure 8:**
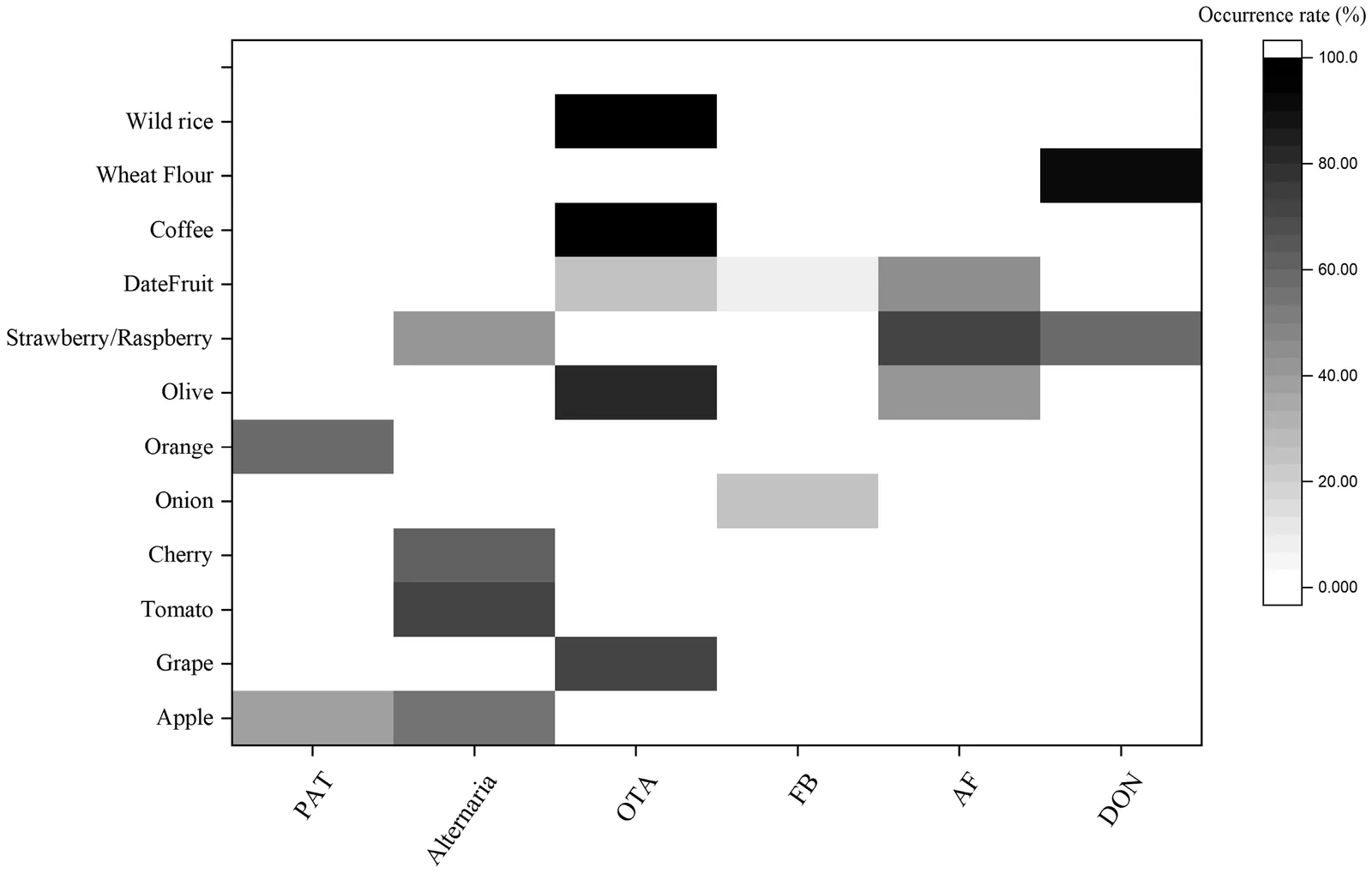
Occurrence rates of major mycotoxins in different agri-food matrices. The color scale represents the percentage of samples exceeding the established legal limits, whenever available. For matrices without defined regulatory limits, all reported occurrence percentages were considered, as these cases may represent higher potential risks due to the absence of legal thresholds.

Figure 8 summarizes the reported occurrence rates of major mycotoxins in these matrices based on the percentage of samples exceeding established legal limits, whenever available. For wild rice, the data corresponds to just two samples analyzed, which limits the representativeness of these results. Overall, these observations highlight the importance of integrating mitigation and safety considerations when applying DES-based extraction systems.

#### 5.2.2. Extraction with DESs

The use of DESs has gained prominence as a green solvent alternative for the valorization of agri-food by-products rich in bioactive compounds. However, most studies overlook the possible presence of mycotoxins, an important aspect given the high susceptibility of these matrices to fungal contamination previously described in the literature. Overall, the most extensively studied DESs include choline chloride (ChCl)-based systems combined with polyols (glycerol (Gly) and ethylene glycol (EG)), organic acids (lactic, citric, oxalic, and malic), carbohydrates, or urea, often containing 10−40% water to reduce viscosity and improve extraction efficiency (Table 3).

In fruit residues from apple, cherry, strawberry, and raspberry, matrices particularly rich in polyphenols, flavonoids, and anthocyanins [114,159,160], ChCl-based DESs have shown outstanding performance. Systems such as ChCl:EG and ChCl:Gly outperformed conventional solvents in apple residues for the extraction of procyanidin B2, epicatechin, quercetin, rutin, gallic acid, and chlorogenic acid [161,162], while oxalic acid-containing systems also produced extracts with high antioxidant activity [163]. In cherries and other red fruits, acid-based DESs (ChCl:malic acid and ChCl:glycolic/oxalic acid) were among the most effective [160,164], particularly when combined with assisted extraction techniques such as microwave or ultrasound treatment. Cocoa bean shells have also been successfully valorized using choline chloride-based DESs. Benítez-Correa et al. evaluated DESs composed of choline chloride combined with lactic acid, glycerol, or ethylene glycol and demonstrated that the addition of water promoted the extraction and diffusion of phenolic compounds. The resulting extracts contained relevant bioactive compounds, including theobromine, caffeine, catechin, and epicatechin, and exhibited antioxidant activity [165].

Tomato by-products (pomace and leaves) have also been efficiently processed using different DES classes. Relatively hydrophobic DESs such as menthol:butyric acid or ethyl acetate:ethyl lactate facilitated the extraction of carotenoids [166,167], while hydrophilic systems (ChCl:lactic acid) enhanced the recovery of phenolics and flavonoids [168,169]. In tomato leaves, the same ChCl:lactic acid DES also showed excellent performance, with good solvent recovery and reusability [170].

Citrus by-products likewise benefit from DES-based extraction, with particularly strong results for peels. In orange peel, ChCl:EG (1:4, 10% (*w*/*w*) H₂O) stood out for phenolic extraction [17], while more recent studies have further demonstrated the effectiveness of different DES formulations. Betaine:Gly (1:2, 20% (*w*/*w*) H₂O) was the most effective system for phenolic recovery from orange peel [171]. In another recent study, ChCl:Gly:citric acid (1:1:1, 25% (*w*/*w*) H₂O) yielded 3700 ± 60.1 µg/mL of total phenolic compounds from orange peel, with hesperidin as the predominant compound [172]. More recently, ChCl:tartaric acid (1:2, 50% H₂O) was identified as the most suitable DES for phenolic recovery from lemon peels, whereas ChCl:glycerol (1:2, 50% H₂O) was selected for lime peel based on the overall bioactivity of the resulting extract [173]. In grapefruit, a lactic acid:glucose DES displayed high selectivity toward flavonoids, achieving yields comparable to ethanol and far exceeding those of water [174].

Although the literature on pomegranate by-products remains limited, ChCl:acetic acid:H_2_O showed strong potential for extracting proteins and bioactive peptides [175]. For polyphenols, however, ethanol/water mixtures remain the most efficient method.

Phenolic-rich matrices derived from olive processing, including pomace, leaves, and virgin olive oil, have proven highly compatible with organic acid- or polyol-based DESs. In olive pomace, systems such as ChCl:citric acid (1:2 or 2:1) yielded high phenolic contents and strong antioxidant activity [176,177], as did lactic acid:glucose [178]. In olive leaves, ChCl:EG (1:2, 43% (*v*/*v*) H_2_O) achieved results comparable to methanol/water, with excellent recovery of oleuropein and flavonoids [179]. For virgin olive oil, DESs such as ChCl:xylitol or ChCl:1,2-propanediol markedly improved the extraction of oleacein, oleocanthal, and other phenolics [180,181]. Although mycotoxin contamination has been documented in olive-derived products, this topic remains largely absent from DES-focused research. 

High extraction efficiencies of ChCl-polyol or ChCl-acid DESs have also been reported for onion skin, eucalyptus leaves, wild rice, date seeds, coffee, and cinnamon. Onion skin produced very high phenolic levels with ChCl:urea:H_2_O [182]; eucalyptus leaves were effectively extracted using ChCl:EG [183]; and in Chinese wild rice, a ChCl:1,4-butanediol system proved effective for phenolics and flavonoids [184]. For date seeds, ChCl:xylose and ChCl:lactic acid performed well [185], while ChCl:Gly and betaine:triethylene glycol were suitable for coffee [186,187]. DESs have also been applied to almond oil extraction, with ChCl:EG (1:2) efficiently recovering ferulic, caffeic, and cinnamic acids [188]. Discarded cinnamon branches, a source of valuable bioactive compounds, yielded up to 41% more cinnamaldehyde and cinnamic acid with ChCl:EG compared with conventional solvents [189]. Furthermore, a recent study developed a method for determining folic acid in fortified flours using the DES TOMAC:isoamyl alcohol (1:4), achieving high recoveries [190].

Taken together, these examples show that some agri-food matrices have been far more extensively explored than others. Most DES formulations and optimization studies have concentrated on polyphenol-rich by-products, particularly those from tomato and grape processing, as well as materials from the olive sector. In contrast, wastes from strawberry, raspberry, cherry, pomegranate, and dates remain underexplored, despite their high antioxidant potential. This uneven distribution highlights clear opportunities for future research, especially in matrices highly susceptible to mycotoxins but rarely assessed in DES-based studies.

Overall, the literature indicates that polar DESs (ChCl with polyols or organic acids) are the most versatile and efficient solvents for extracting polyphenols, flavonoids, and alkaloids, while hydrophobic DESs favor carotenoid extraction. Moderate water addition (10−40%) generally improves performance without compromising eutectic behavior. The major limitation across studies is the lack of safety assessments, particularly regarding co-extraction of mycotoxins and cytotoxicity. Thus, although DESs stand out as a promising green extraction strategy, their industrial application requires studies that jointly address efficiency, safety, and traceability of fungal contamination.

**Table 3:** Summary of DES-based extraction studies on agri-food by-products and products, together with mycotoxin occurrence (independent literature unrelated to the DES extraction studies).

Agri-Food By-Product		DES Composition (Molar Ratio)	Extraction Method		Bioactive Compounds/Extraction Outcome	Toxicity Assessment (Pre and Post)	Ref.	Mycotoxins Reported for the Matrix (Independent Literature) *
Pomace	Apple	ChCl:oxalic acid (1:1, 30% (*v*/*v*) H_2_O)	UAE, 60 °C, 45 min, solid/liquid ratio: 1:10 g/mL		TPC: 9.97 mg GAE/g FW HPLC (µg/g dw): phlorizin: 448.7; chlorogenic acid: 388.3; epicatechin: 113.7; rutin: 235.3; gallic acid: 87.3 Antioxidant activity: DPPH: 76%; ABTS: 85%; •OH: 71%	Not tested	[163]	PAT *Alternaria* toxins
		ChCl:Gly (1:2) and ChCl:lactic acid (1:3), both with 30% (*v*/*v*) H_2_O	UAE, 40 °C, 40 min, solid/liquid ratio: 1:30 g/g		TPC: 5.6 mg GAE/g dw TFC: 5.4 mg QE/g dw HPLC (µg/g dw): quercetin: 1628; chlorogenic acid: 1181; gallic acid: 972; rutin: 301 Antioxidant activity: DPPH: 70%		[162]	
		ChCl:EG (1:4, 25% (*w*/*w*) H_2_O)	UAE, 60 °C, 6 h, solid/liquid ratio: 1:10 g/g		Qualitative analysis: procyanidin B2, chlorogenic acid, epicatechin, vanillin, phloridzin, quercetin derivatives Antioxidant activity: DPPH: 80%	Cytotoxicity: extract considered safe at low concentrations (<50 µg/mL)	[161]	
	Grape	ChCl:citric acid (2:1, 30% (*v*/*v*) H_2_O)	UMAE, 10 min, solid/liquid ratio: 1:10 g/mL		TAC: 1.77 mg/g dw	Not tested	[99]	OTA
		Betaine:citric acid (40% (*w*/*w*) H_2_O)	Magnetic stirring, RT, 24 h, solid/liquid ratio: 1:40 g/g		TPC: 26.02 ± 1.16 mg GAE /mL; TAC: 9.07 ± 0.05 mg EMV/mL; TFC: 0.46 ± 0.01 mg EC/mL; malvidin: 56.66 µg/mL	LDH assay in 3D HaCaT keratinocytes; non-cytotoxic at ≥1:100 *v*/*v*	[100]	
		Betaine:lactic acid (1:2, 5% (*v*/*v*) H_2_O)	Water bath immersion, 65 °C, 60 min, solid/liquid ratio: 1:10 g/mL		Composition (µg/mL): gallic acid: 792.4 µg/mL; caffeic acid: 164.5 µg/mL; catechin: 511.3 µg/mL Anthocyanins: ~19%	No toxicity was observed	[101]	
		ChCl:lactic acid (1:2, 25% (*w*/*w*) H_2_O)	UAE, 60 °C, 60 min, solid/liquid ratio: 1:20 g/mL		Composition (µg/g dw): Dp3-O-Glu (73,381.26); Pd3-O-Glu (9547.72); petunidin (2403.89); delphinidin (1289.47) Antioxidant activity: DPPH: 87%; ABTS: 92%; FRAP: 4 µmol/mL	Not tested	[102]	
		Gly:glucose (1:1, 25% (*w*/*w*) H_2_O)	Orbital shaking, 44 °C, solid/liquid ratio: 5 g/30 mL		Enhanced phenolic extraction compared with water; catechin, gallic acid, syringic acid, luteolin-7-O-glucoside, quercetin-3-O-glucuronide and other phenolics identified	Mycotoxin safety assessed	[55]	
	Tomato	Menthol:butyric acid (1:2)	Shaking incubator, 65 °C, 50 min, solid/liquid ratio: 1:10 g/mL		204 µg/g dw total carotenoids 24 µg/g dw β-carotene 52 µg/g dw lycopene	Not tested	[166]	*Alternaria* mycotoxins
		Ethyl acetate:ethyl lactate (30:70 % (*v*/*v*))	UAE, 60 °C, 20 min, solid/liquid ratio: 1:20 g/mL		3950.08 µg/g dw β-carotene 75.86 µg/g dw lycopene		[167]	
		ChCl:lactic acid (1:2, 10% (*v*/*v*) H_2_O)	Stirring incubator, 65 °C, 60 min, solid/liquid ratio 1:100 g/mL		TPC: 51.75 ± 1.15 mg GAE/g TFC: 17.31 ± 1.20 mg QE/g Chlorogenic acid: 52.33 µg/g dw DPPH: 77%		[168]	
		ChCl:lactic acid (1:2 *v*/*w*, 10% (*w*/*w*) H_2_O)	UAE, 65 °C, 60 min, solid/liquid ratio: 1:20 g/mL		Composition (µg/g dw): gallic acid (14.4 ± 0.80), chlorogenic acid (52.33 ± 0.80), caffeic acid (5.03 ± 0.37), ferulic acid (1.51 ± 0.013), quercetin (1.47 ± 0.11) Antioxidant activity: 0.511 mg AAE eq/g		[169]	
	Olive	ChCl:citric acid (1:2, 20% (*v*/*v*) H_2_O)	Homogenization, 60 °C, 30 min, solid/liquid ratio: 1:20 g/mL		Qualitative analysis: hydroxytyrosol, oleuropein, caffeic acid, vanillin, rutin, luteolin TPC: 34.08 mg GAE/g dw Antioxidant activity (DPPH, IC₅₀): 5.11 ± 0.85 g dw/g DPPH		[176]	Capacity to adsorb AFB1, OTA, and ZEN
		ChCl:citric acid (2:1, 30% (*v*/*v*) H_2_O)	UMAE, 10 min, solid/liquid ratio: 1:20 g/mL		Composition (µg/g dw): gallic acid (43.79 ± 7.15), hydroxytyrosol (195.61 ± 20.06), tyrosol (136.67 ± 15.38), vanillic acid (52.06 ± 3.19), vanillin (135.21 ± 16.07), pinoresinol (48.19 ± 2.20), catechin (34.46 ± 0.75) TPC: 646 ± 65 mg/kg dw ORAC: 453.10 ± 51.09 µmol Trolox eq/g dw	Significant antiproliferative activity in tumor cells (HeLa and MCF-7), with viability reduced to ~12–38%	[177]	
		Lactic acid:glucose (5:1, 15% (*v*/*v*) H_2_O)	UAE, 40 °C, 60 min, solid/liquid ratio: 1:13 g/mL		Composition (µg/g dw): luteolin (453,700), apigenin (79,300), hydroxytyrosol (111,000), ferulic acid (8200), rutin (12,600), tyrosol (1360), and caffeic acid (3230)	Not tested	[178]	
	Cherry	ChCl:malic acid (1:1, 30% (*w*/*w*) H_2_O)	MAE, 50 °C, 5 min, solid/liquid ratio: 1:10 g/mL		TPC: 68 mg GAE/g dw TAC: 29 mg C3G/g dw TFC: 6 mg CE/g dw TPA: 18 mg CAE/g dw		[164]	*Alternaria* mycotoxins
Peel/ skin	Grape	ChCl:oxalic acid (1:1, 25% (*w*/*w*) H_2_O)	UAE, 65 °C, 50 min, solid/liquid ratio: 1:10 g/mL		Total anthocyanins: ~30 mg/g dw		[105]	OTA
		Citric acid:maltose (4:1, 24% (*w*/*w*) H_2_O)	UAE, RT, 9 min, solid/liquid ratio: 1:8.3 g/mL		TAC: 63.36 mg C3,5-dg eq./g dw DPPH: 59%		[104]	
	Onion	ChCl:urea:H_2_O (1:2:4)	MAE, 60 °C, 120 min, solid/liquid ratio: 1:50 g/mL		Quercetin (6180 µg/g dw), kaempferol (350 µg/g dw), myricetin (110 µg/g dw) TPC: 222.97 mg GAE/g dw DPPH: 76% FRAP: 1457.19 µmol AAE eq/g		[182]	Fumonisins (FB1, FB2, FB3 and FB4), beauvericin, moniliformin
	Pomegranate	ChCl:acetic acid:H_2_O (1:1:10)	HIFU, 11 min, solid/liquid ratio: 1:33 g/mL		20 ± 1 mg/g protein dw; high antihypertensive activity (IC_50_ ≈ 23–28 µg/mL)		[175]	Potential risk (mycotoxigenic fungi identified)
	Orange	ChCl:EG (1:4, 10% (*w*/*w*) H_2_O)	SLE, 60 °C, 100 min, solid/liquid ratio: 1:10 g/mL		TPC: 3.61 mg GAE/g dw DPPH IC_50_: 30.6 µg/mL		[17]	PAT
		Betaine:Gly (1:2, 20% (*w*/*w*) H₂O)	UAE, 30 °C, 60 min, NADES/peel ratio: 30 g/g		TPC: 1130.86 ± 30.68 mg GAE/100 g; DPPH inhibition: 38.27 ± 2.43%; ABTS: 2977 ± 224 µmol Trolox eq/100 g; hesperidin, p-coumaric acid, naringin and rutin also identified		[171]	
		ChCl:Gly:citric acid (1:1:1, 25% (*w*/*w*) H₂O)	Stirring, 45 °C, 30 min, solid/liquid ratio: 1:10 g/mL		TPC: 3.7 ± 0.060 mg/mL Hesperidin: 8–94% of total polyphenols		[172]	
	Lemon	ChCl:tartaric acid (1:2, 50% H₂O)	UAE, RT, 50 min, solid/liquid ratio: 0.2 g/10 mL (1:50 g/mL)		TPC: 0.766 ± 0.012 mg GAE/mL Hesperidin was the major phenolic compound identified		[173]	__________
	Lime	ChCl:glycerol (1:2, 50% H₂O)	UAE, RT, 50 min, solid/liquid ratio: 0.2 g/10 mL (1:50 g/mL)		TPC: 0.420 ± 0.020 mg GAE/mL Hesperidin was the major phenolic compound identified			__________
	Grapefruit	Lactic acid:glucose 30% (*w*/*w*) H_2_O	HVED + SLE, 50 °C, 2 h, solid/liquid ratio: 1:20 g/mL		Naringin: 1864 µg/100 g dw TPC: até 1.930 mg/100 g dw		[174]	__________
Leaf	Olive	ChCl:ethylene glycol (1:2, 43% (*v*/*v*) H_2_O)	MAE, 79.6 °C, 16.7 min, solid/liquid ratio: 1:50 g/mL		TPC: 25–31 mg GAE/g dw A total of 48 phenolic compounds were identified, oleuropein (12 mg/g) and flavonoids stood out	Not tested	[179]	AFs, ZEN, DON, OTA
	Tomato	ChCl:lactic acid (1:2)	Mechanical Agitation 70 °C, 2 h, solid/liquid ratio: 1:15 g/mL		Composition (μg/g dw): rutin (6236 ± 42), hesperidin (597.5 ± 12), chlorogenic acid (320.4 ± 6); other compounds in lower amounts (quercetin, caffeic acid, etc.) TPC: 13.5 mg GAE/g TFC: 2.5 mg QE/g		[170]	__________
	Eucalyptus	ChCl:EG (1:2, 20% (*v*/*v*) H_2_O)	Orbital shaking, 50 °C, 60 min, solid/liquid ratio: 1:10 g/mL		TPC: 69.9 mg GAE/g dw TFC: 45.4 mg RE/g dw DPPH: 68.0 mg Trolox eq/g dw FRAP: 66.3 mg Trolox eq/g dw ABTS: 89.9 mg Trolox eq/g dw		[183]	__________
Wine lees		ChCl:malic acid (1:1, 35% (*w*/*w*) H_2_O)	UAE, 35 °C, 30.6 min, solid/liquid ratio: 1:10 g/mL		TAC: 6.550 mg/g dw 3660 µg/g dw of anthocyanin-3-O-monoglucosides; 1570 µg/g dw of anthocyanin-3-(6-O-p-coumaroyl) monoglucosides	Not tested	[113]	Capacity to adsorb OTA
Strawberry and raspberry waste		ChCl:glycolic acid:oxalic acid (1:1.7:0.3)	Stirring, RT, 15 min, solid/liquid ratio: 1:1 g/mL		Strawberry (dw): TPC: 5.791 mg GAE/g Anthocyanins: 0.028 mg/g Sugars: 82,230 µg/g DPPH: 2.39 ± 0.15 Trolox eq/g dw Raspberry (dw): TPC: 49.644 mg EAG/g Anthocyanins: 1280 µg/100 g Sugars: 23,660 µg DPPH: 2.07 ± 0.02 TE/g		[160]	AFs, DON, *Alternaria* mycotoxins
By-products of date fruit		ChCl:lactic acid (1:2, 37% (*v*/*v*) H_2_O)	MAE, 80 °C, solid/liquid ratio: 1:10 g/mL	5 min	TPC: 147.92 mg GAE/g DPPH: 503 mM Trolox eq/g dw FRAP: 771 mM Trolox eq/g dw		[185]	OTA, FB2, AFB1, AFB2, enniatin B
		ChCl:xylose (1:1, 50% (*v*/*v*) H_2_O)		20 min	TPC: 234.65 mg GAE/g dw DPPH: 396 mM Trolox eq/g dw FRAP: 734 mM Trolox eq /g dw			
Cinnamon twig residue		ChCl:EG (1:6, 30% (*v*/*v*) H_2_O)	MAE, 60 °C, 60 min, solid/liquid ratio: 1:60 g/mL		Cinnamic acid: 590 µg/g dw Cinnamaldehyde: 12,290 µg/g dw		[189]	Potential risk in commercial cinnamon
Coffee by-products	Coffee pulp	ChCl:Gly (1:3, 30% (*v*/*v*) H_2_O)	Magnetic stirring, 55 °C, 120 min, solid/liquid ratio: 1:47 g/mL		5190 µg caffeine/g dw DPPH: 82 µmol Trolox eq/g extract		[186]	__________
	Spent coffee grounds	Betaine:triethylene glycol (1:2, 30% (*v*/*v*) H_2_O)	UAE, 65 °C, 20 min, solid/liquid ratio: 1:15 g/mL		4.64 mg CGAs/g dw		[187]	
Products								
Olive oil		ChCl:1,2-propanediol (1:1)	Liquid-liquid extraction, 40 °C, 1 h, vortex 1 min every 15 min, oil/DES ratio: 1:1 g/g		Oleacein and oleocanthal were predominant, with high efficiency also observed for other phenolic compounds	Not tested	[180]	AFB1, OTA
		ChCl:xylitol:H_2_O (2:1:3)	Water bath agitation, 40 °C, 1 h, oil/DES ratio: 1:1 g/g		0.555 mg/g olive oil (total phenolic content quantified by HPLC-DAD)		[181]	
Cinnamon Oil		ChCl:EG (1:2)	UA-DLLME, 30 °C, 5 min, oil + hexane (4 mL)/DES (0.05 mL) ≈ 80:1 *v*/*v*		Ferulic acid: 36 μg/L; cinnamic acid: 270 μg/L; caffeic acid: low concentration		[188]	__________
Wheat flour		TOMAC:isoamyl alcohol (1:4)	VA-DLLME, RT, 1 min vortex, MeOH/DES ratio: 1.66:1 *v*/*v*		Folic acid recovery: 91.6–99.7%		[190]	DON, ZEN, T-2, enniatin
Chinese wild rice		ChCl:1,4-butanediol (1:6, 23% (*w*/*w*) H_2_O)	UAE, 51.2 °C, 10 min, solid/liquid ratio: 37 mg/mL		TFC: 9.30 mg QE/g Total phenolics (UPLC-QqQ-MS): 0.267 mg/g Major compounds (µg/g): catechin (23.51), quercetin (21.12), ferulic acid (114.84)		[184]	OTA

* Reported mycotoxin occurrence refers to independent literature on the corresponding matrix and does not imply contamination of the specific extracts unless directly assessed in the same study.

## 6. Current Research Gaps and Challenges

Despite the increasing number of studies on enzymatic mycotoxin mitigation and the growing application of DESs in biocatalysis, several important limitations remain. One of the most significant gaps is the strong reliance on simplified model systems. As discussed throughout this review, enzymatic degradation is frequently evaluated in buffer solutions, where high efficiencies are often achieved, but these results do not necessarily translate to real and complex food matrices. The presence of phenolic compounds, proteins, and other matrix components may interfere with enzyme activity, leading to reduced performance and limiting practical applicability. Differences in enzyme source and dose, mycotoxin concentration, reaction conditions, and analytical approaches further limit direct comparison between studies.

Another relevant limitation is the scarce information regarding the interaction between lipases and DESs in the specific context of mycotoxin degradation. Although DESs have demonstrated the ability to maintain or even enhance enzymatic activity in other reactions, their effect on degradation pathways, enzyme stability, and substrate accessibility in mycotoxin systems remains poorly understood. In particular, the role of DES composition, polarity, and water content requires systematic investigation.

Furthermore, safety aspects are still largely overlooked. In many cases, studies assume that enzymatic degradation leads to less toxic products, but comprehensive identification and toxicological assessment of transformation products are often lacking. This represents a critical gap, especially when considering applications in food systems.

Analytical reliability should also be considered when interpreting mycotoxin degradation studies. A decrease in the parent toxin alone may not be sufficient to confirm degradation, particularly in complex matrices. Analytical validation, assessment of matrix effects, and identification of relevant transformation products should therefore be considered, together with modified or masked mycotoxins where relevant.

The sustainability of DESs also requires case-by-case evaluation. Although NADESs are commonly formulated from naturally occurring and often food-grade components and generally present favorable safety and environmental profiles, these properties depend on the specific formulation. Their overall sustainability should therefore also consider biodegradability, viscosity, water and energy requirements, solvent and enzyme recovery, extract purification, scale-up feasibility, regulatory acceptance, and life-cycle performance.

Finally, the combination of mycotoxin mitigation and the valorization of agri-food by-products remains underexplored. Although DESs are widely applied for the extraction of bioactive compounds, their integration with enzymatic mycotoxin mitigation strategies has not yet been sufficiently investigated, representing an important opportunity for future research.

## 7. Conclusions and Future Perspectives

This review highlights that lipases remain an underexplored strategy for mycotoxin mitigation, particularly in real and complex food matrices, where enzymatic performance is still insufficiently understood. A key finding is that the reaction medium plays a decisive role in determining enzymatic efficiency, and results obtained in simplified buffer systems cannot be reliably extrapolated to real agri-food environments. This discrepancy underscores the urgent need for studies conducted under realistic processing conditions.

DESs emerge as versatile and tunable green media capable of modulating enzyme performance while enabling the sustainable extraction of bioactive compounds from agri-food by-products. However, despite the rapid expansion of DES-based research, their application in enzymatic mycotoxin degradation remains largely unexplored. Although direct evidence has recently been reported for OTA degradation by porcine pancreatic lipase in glycerol-based DES-containing systems, this evidence is currently limited to a single study. Broader DES–lipase applications therefore remain an emerging research hypothesis that requires validation across other mycotoxins, lipases, DES formulations, and contaminated food matrices. The combination of DESs with immobilized lipases should also be assessed in terms of retained activity, operational stability, reusability, resistance to the selected solvent system, and performance in complex matrices.

At the same time, the valorization of mycotoxin-prone by-products using DESs has advanced significantly, but critical safety aspects—particularly the co-extraction, transformation, or persistence of mycotoxins—are still largely overlooked. Integrated strategies combining enzymatic degradation with green extraction technologies should therefore include direct quantification of parent mycotoxins in both recovered fractions and residual matrices, identification and toxicological assessment of relevant transformation products, and verification that contaminants are not concentrated during extraction.

From an industrial perspective, the scalability and economic feasibility of these systems must also be addressed. Factors such as enzyme cost, stability under processing conditions, and solvent recyclability will play a decisive role in determining their practical implementation. Enzyme immobilization and rational DES design may contribute to improving process robustness and reducing operational costs.

Ultimately, advancing this field will depend on interdisciplinary efforts that bridge enzymology, green chemistry, food safety, and process engineering. Demonstrating safety, reproducibility, and technical feasibility in real contaminated matrices will be essential for translating current laboratory findings into viable industrial applications.

## Figures and Tables

**Table 1:** Comparison of the scope of representative previous reviews and the present review. ✓ = central/substantial focus of the review; ◐ = addressed, but not a main focus; — = not addressed to a significant extent.

Review	Enzymatic Mycotoxin Degradation	Lipases/Enzymes in DESs	Enzyme/Lipase Engineering *	DES-Based Bioactive Extraction	Agri-Food By-Product Valorization	Mycotoxin Safety in By-Products	Mitigation–Valorization Framework
[6]	✓	—	◐	—	—	—	—
[20]	✓	—	✓	—	—	—	—
[21]	—	✓	—	—	—	—	—
[22]	—	✓	◐	—	—	—	—
[23]	—	✓	—	◐	—	—	—
[24]	—	—	✓	—	—	—	—
[25,26,27]	—	—	—	✓	✓	—	—
[28]	—	—	—	—	◐	✓	—
[29]	—	—	—	—	✓	✓	✓ ^1^
[30]	◐	—	◐	—	✓	—	◐
Present review	✓	✓	✓	✓	✓	✓	✓

* Includes enzyme immobilization and/or modification/engineering strategies. ^1^ Integration refers to biological mycotoxin mitigation combined with energy and nutrient recovery through anaerobic digestion and composting, rather than DES-based bioactive compound recovery.

## Data Availability

No new data were created or analyzed in this study. Data sharing is not applicable to this article.

## Abbreviations

The following abbreviations are used in this manuscript:

AAE	Ascorbic acid equivalents
ABTS	2,2′-Azino-bis(3-ethylbenzothiazoline-6-sulfonic acid)
AFs	Aflatoxins
AFB1	Aflatoxin B1
AFB2	Aflatoxin B2
AFM1	Aflatoxin M1
AME	Alternariol monomethyl ether
AOH	Alternariol
C3G	Cyanidin-3-glucoside
C3,5-dg	Cyanidin-3,5-diglucoside
CAE	Caffeic acid equivalents
CALB	*Candida antarctica* lipase B
CBMA	Carboxybutyl methacrylate
CE	Catechin equivalents
CGAs	Chlorogenic acids
ChCl	Choline chloride
CLEA	Cross-linked enzyme aggregate
CPA	Carboxypeptidase A
DES	Deep eutectic solvent
DMAPA	Dimethylaminopropyl acrylate
DON	Deoxynivalenol
DPPH	2,2-Diphenyl-1-picrylhydrazyl
EG	Ethylene glycol
EFSA	European Food Safety Authority
EU	European Union
FB1	Fumonisin B1
FB2	Fumonisin B2
FB3	Fumonisin B3
FB4	Fumonisin B4
FRAP	Ferric reducing antioxidant power
FUM	Fumonisin
GAE	Gallic acid equivalents
Gly	Glycerol
HBA	Hydrogen bond acceptor
HBD	Hydrogen bond donor
HIFU	High-intensity focused ultrasound
HPLC	High-performance liquid chromatography
HPLC-DAD	High-performance liquid chromatography with diode-array detection
HVED	High-voltage electrical discharge
IC50	Half-maximal inhibitory concentration
LC-MS²	Liquid chromatography-tandem mass spectrometry
LDH	Lactate dehydrogenase
MAE	Microwave-assisted extraction
MOF	Metal-organic framework
NIBMA	N-Isobutyl methacrylate
ORAC	Oxygen radical absorbance capacity
OTA	Ochratoxin A
PAT	Patulin
PBS	Phosphate-buffered saline
PEG	Polyethylene glycol
PEI	Polyethyleneimine
PLA	Polylactic acid
PPL	Porcine pancreatic lipase
PVA	Poly(vinyl alcohol)
QE	Quercetin equivalents
RE	Rutin equivalents
RT	Room temperature
SLE	Solid-liquid extraction
TAC	Total anthocyanin content
TBAB	Tetrabutylammonium bromide
TBMA	*tert*-Butyl methacrylate
TeA	Tenuazonic acid
TEN	Tentoxin
TFC	Total flavonoid content
TLL	*Thermomyces lanuginosus* lipase
TOMAC	Trioctylmethylammonium chloride
TPA	Total phenolic acids
TPC	Total phenolic content
UA-DLLME	Ultrasound-assisted dispersive liquid–liquid microextraction
UAE	Ultrasound-assisted extraction
UMAE	Ultrasound–microwave-assisted extraction
UPLC-QqQ-MS	Ultra-performance liquid chromatography coupled to triple-quadrupole mass spectrometry
VA-DLLME	Vortex-assisted dispersive liquid–liquid microextraction
ZEN	Zearalenone
ZHD	Zearalenone hydrolase

## References

[1] Alshannaq A., Yu J.-H. (2017). Occurrence, Toxicity, and Analysis of Major Mycotoxins in Food. Int. J. Environ. Res. Public Health.

[2] EFSA Panel on Contaminants in Food Chain (CONTAM) (2011). Scientific Opinion on the risks for animal and public health related to the presence ofAlternariatoxins in feed and food. EFSA J..

[3] Palumbo R., Crisci A., Venâncio A., Cortiñas Abrahantes J., Dorne J.-L., Battilani P., Toscano P. (2020). Occurrence and co-occurrence of mycotoxins in cereal-based feed and food. Microorganisms.

[4] Nazareth T.d.M., Pérez E.S., Luz C., Meca G., Quiles J.M. (2024). Comprehensive Review of Aflatoxin and Ochratoxin A Dynamics: Emergence, Toxicological Impact, and Advanced Control Strategies. Foods.

[5] Silva A.S., Brites C., Pouca A.V., Barbosa J., Freitas A. (2019). UHPLC-ToF-MS method for determination of multi-mycotoxins in maize: Development and validation. Curr. Res. Food Sci..

[6] Liu M., Zhang X., Luan H., Zhang Y., Xu W., Feng W., Song P. (2024). Bioenzymatic detoxification of mycotoxins. Front. Microbiol..

[7] Karlovsky P., Suman M., Berthiller F., De Meester J., Eisenbrand G., Perrin I., Oswald I.P., Speijers G., Chiodini A., Recker T. (2016). Impact of food processing and detoxification treatments on mycotoxin contamination. Mycotoxin Res..

[8] Abraham N., Chan E.T.S., Zhou T., Seah S.Y.K. (2022). Microbial detoxification of mycotoxins in food. Front. Microbiol..

[9] Liu M., Zhao L., Gong G., Zhang L., Shi L., Dai J., Han Y., Wu Y., Khalil M.M., Sun L. (2022). Invited review: Remediation strategies for mycotoxin control in feed. J. Anim. Sci. Biotechnol..

[10] (2023). Commission Regulation (EU) 2023/915 of 25 April 2023 on Maximum Levels for Certain Contaminants in Food and Repealing Regulation (EC) No 1881/2006. Off. J. Eur. Union.

[11] (2015). Commission Regulation (EU) 2015/786 of 19 May 2015 Defining Acceptability Criteria for Detoxification Processes Applied to Products Intended for Animal Feed as Provided for in Directive 2002/32/EC of the European Parliament and of the Council. Off. J. Eur. Union.

[12] Strong P.J., Claus H. (2011). Laccase: A Review of Its Past and Its Future in Bioremediation. Crit. Rev. Environ. Sci. Technol..

[13] Loi M., Fanelli F., Liuzzi V.C., Logrieco A.F., Mule G. (2017). Mycotoxin biotransformation by native and commercial enzymes: Present and future perspectives. Toxins.

[14] Upadhaya S.D., Park M.A., Ha J.K. (2010). Mycotoxins and Their Biotransformation in the Rumen: A Review. Asian-Australas. J. Anim. Sci..

[15] Andualema B., Gessesse A. (2012). Microbial Lipases and Their Industrial Applications: Review. Biotechnology.

[16] Wang S., Cai R., Liu X., Qi L., Wang L., Yue T., Yuan Y., Wang Z. (2023). The detoxification of ochratoxin A in wine and grape juice by different enzymes and evaluation of their effects on the quality. Efood.

[17] Ozturk B., Parkinson C., Gonzalez-Miquel M. (2018). Extraction of polyphenolic antioxidants from orange peel waste using deep eutectic solvents. Sep. Purif. Technol..

[18] Castellet-Viciano L., Hernández-Chover V., Bellver-Domingo Á., Hernández-Sancho F. (2025). The Role of Circular Economy Strategies in Promoting Sustainability in the Agri-Food Sector: Insights from the Valencian Community. Appl. Sci..

[19] Ahmad T., Esposito F., Cirillo T. (2024). Valorization of agro-food by-products: Advancing sustainability and sustainable development goals 2030 through functional compounds recovery. Food Biosci..

[20] Wang Y., Chen Y., Jiang L., Huang H. (2022). Improvement of the enzymatic detoxification activity towards mycotoxins through structure-based engineering. Biotechnol. Adv..

[21] Pätzold M., Siebenhaller S., Kara S., Liese A., Syldatk C., Holtmann D. (2019). Deep Eutectic Solvents as Efficient Solvents in Biocatalysis. Trends Biotechnol..

[22] Tan J.-N., Dou Y. (2020). Deep eutectic solvents for biocatalytic transformations: Focused lipase-catalyzed organic reactions. Appl. Microbiol. Biotechnol..

[23] Buzatu A.R., Todea A., Peter F., Boeriu C.G. (2024). The Role of Reactive Natural Deep Eutectic Solvents in Sustainable Biocatalysis. ChemCatChem.

[24] Noro J., Cavaco-Paulo A., Silva C. (2022). Chemical modification of lipases: A powerful tool for activity improvement. Biotechnol. J..

[25] Ristivojević P., Ristivojević M.K., Stanković D., Cvijetić I. (2024). Advances in Extracting Bioactive Compounds from Food and Agricultural Waste and By-Products Using Natural Deep Eutectic Solvents: A Circular Economy Perspective. Molecules.

[26] Mavai S., Bains A., Sridhar K., Chawla P., Sharma M. (2024). Emerging deep eutectic solvents for food waste valorization to achieve sustainable development goals: Bioactive extractions and food applications. Food Chem..

[27] Gomez-Urios C., Paños-Huertas M., Castillo-Esteve I., Cortes C., Blesa J., Esteve M.J. (2026). From green extraction to clean-label formulation: Natural deep eutectic solvents as multifunctional extraction–formulation systems for agri-food valorization. Food Res. Int..

[28] Lopes P., Sobral M.M.C., Lopes G.R., Martins Z.E., Passos C.P., Petronilho S., Ferreira I.M.P.L.V.O. (2023). Mycotoxins’ Prevalence in Food Industry By-Products: A Systematic Review. Toxins.

[29] Cucina M., Tacconi C. (2022). Recovery of Energy and Nutrients from Mycotoxin-Contaminated Food Products through Biological Treatments in a Circular Economy Perspective: A Review. Agronomy.

[30] Sheng L., Gao S., Yang Y., Yan H., Ma W., Sun X. (2026). Synthetic Biology Approaches in Reducing Mycotoxin Contamination and Enhancing Food System Sustainability. Annu. Rev. Food Sci. Technol..

[31] Liu B., Peng X., Meng X. (2018). Effective Biodegradation of Mycotoxin Patulin by Porcine Pancreatic Lipase. Front. Microbiol..

[32] Liu L., Xie M., Wei D. (2022). Biological Detoxification of Mycotoxins: Current Status and Future Advances. Int. J. Mol. Sci..

[33] Stander M.A., Bornscheuer U.T., Henke E., Steyn P.S. (2000). Screening of Commercial Hydrolases for the Degradation of Ochratoxin A. J. Agric. Food Chem..

[34] Kholif A.M.M., Fouad M.T., A El-Desouky T. (2022). Evaluation of enzymatic degradation of ochratoxin A by protease and lipase produced by lactobacillus isolated from dairy products. Int. J. Front. Biol. Pharm. Res..

[35] Kokkinou M., Theodorou L.G., Papamichael E.M. (2012). Aspects on the catalysis of lipase from porcine pancreas (type VI-s) in aqueous media: Development of ion-pairs. Braz. Arch. Biol. Technol..

[36] Borrelli G.M., Trono D. (2015). Recombinant Lipases and Phospholipases and Their Use as Biocatalysts for Industrial Applications. Int. J. Mol. Sci..

[37] Mendes A.A., Oliveira P.C., de Castro H.F. (2012). Properties and biotechnological applications of porcine pancreatic lipase. J. Mol. Catal. B Enzym..

[38] Chen W., Li C., Zhang B., Zhou Z., Shen Y., Liao X., Yang J., Wang Y., Li X., Li Y. (2018). Advances in Biodetoxification of Ochratoxin A-A Review of the Past Five Decades. Front. Microbiol..

[39] Martinez-Gonzalez A.I., Alvarez-Parrilla E., Díaz-Sánchez Á.G., de la Rosa L.A., Núñez-Gastélum J.A., Vazquez-Flores A.A., Gonzalez-Aguilar G.A. (2017). In Vitro Inhibition of Pancreatic Lipase by Polyphenols: A Kinetic, Fluorescence Spectroscopy and Molecular Docking Study. Food Technol. Biotechnol..

[40] Serea D., Condurache N.N., Aprodu I., Constantin O.E., Bahrim G.-E., Stănciuc N., Stanciu S., Rapeanu G. (2022). Thermal Stability and Inhibitory Action of Red Grape Skin Phytochemicals against Enzymes Associated with Metabolic Syndrome. Antioxidants.

[41] Khan M.U., Yu P., Wu Y., Chen Z., Kong L., Farid A., Cui J., Yang J. (2025). Comprehensive review of enzymes (protease, lipase) in milk: Impact on storage quality, detection methods, and control strategies. Compr. Rev. Food Sci. Food Saf..

[42] Reis P.M., Raab T.W., Chuat J.Y., Leser M.E., Miller R., Watzke H.J., Holmberg K. (2008). Influence of Surfactants on Lipase Fat Digestion in a Model Gastro-intestinal System. Food Biophys..

[43] Al-Maqdi K.A., Elmerhi N., Athamneh K., Bilal M., Alzamly A., Ashraf S.S., Shah I. (2021). Challenges and Recent Advances in Enzyme-Mediated Wastewater Remediation—A Review. Nanomaterials.

[44] El-Desouky T.A., Kholif A.M.M. (2023). Degradation of Aflatoxin M1 by Lipase and Protease in Buffer Solution and Yoghurt. Open Biotechnol. J..

[45] Xiao Y., Liu B., Wang Z., Han C., Meng X., Zhang F. (2019). Effective degradation of the mycotoxin patulin in pear juice by porcine pancreatic lipase. Food Chem. Toxicol..

[46] Chang X., Liu H., Sun J., Wang J., Zhao C., Zhang W., Zhang J., Sun C. (2020). Zearalenone Removal from Corn Oil by an Enzymatic Strategy. Toxins.

[47] Pradanas-González F., Álvarez-Rivera G., Benito-Peña E., Navarro-Villoslada F., Cifuentes A., Herrero M., Moreno-Bondi M.C. (2021). Mycotoxin extraction from edible insects with natural deep eutectic solvents: A green alternative to conventional methods. J. Chromatogr. A.

[48] Kolawole O., Thammakhet-Buranachai C., Petchkongkaew A., Sooksimuang T., Elliott C., Karoonuthaisiri N. (2024). Evaluating the green credentials and performance of deep eutectic solvents in the extraction of antibiotics and mycotoxins in foods. Green Chem. Lett. Rev..

[49] Luque-Uría Á., González F.P., Navarro-Villoslada F., Benito-Peña E. (2025). Natural Deep Eutectic Solvents as a Green Alternative for Extracting Contaminants from Food Samples: A Review. J. Anal. Test..

[50] Noro J., Cabo J., Freitas D.S., Roque C.S., de Castro M., Cavaco-Paulo A., Silva C. (2023). Deep Eutectic Solvents as Suitable Solvents for Lipase-Catalyzed Transesterification Reactions. ChemSusChem.

[51] Freitas D.S., Quesado V., Rocha D., Noro J., Martins M., Cavaco-Paulo A., Silva C. (2023). Lipase-Catalysed Polymerization of Eutectic Mixtures. ChemSusChem.

[52] Nian B., Cao C., Liu Y. (2020). How *Candida antarctica* lipase B can be activated in natural deep eutectic solvents: Experimental and molecular dynamics studies. J. Chem. Technol. Biotechnol..

[53] Liu C., Shi J. (2025). Understanding Lipase-Deep Eutectic Solvent Interactions Towards Biocatalytic Esterification. Catalysts.

[54] Nieto S., Bernal J.M., Villa R., Garcia-Verdugo E., Donaire A., Lozano P. (2023). Sustainable Setups for the Biocatalytic Production and Scale-Up of Panthenyl Monoacyl Esters under Solvent-Free Conditions. ACS Sustain. Chem. Eng..

[55] Santos J., Rocha D., Venâncio A., Silva C. (2025). Glycerol-Based Deep Eutectic Mixtures for Grape Pomace Waste Valorization and Ochratoxin A Degradation. ACS Sustain. Resour. Manag..

[56] Robescu M.S., Bavaro T. (2025). A Comprehensive Guide to Enzyme Immobilization: All You Need to Know. Molecules.

[57] Rana H., Sharma A., Dutta S., Goswami S. (2022). Recent Approaches on the Application of Agro Waste Derived Biocomposites as Green Support Matrix for Enzyme Immobilization. J. Polym. Environ..

[58] Shuai W., Das R.K., Naghdi M., Brar S.K., Verma M. (2016). A review on the important aspects of lipase immobilization on nanomaterials. Biotechnol. Appl. Biochem..

[59] Guimarães J.R., Oliveira K.S.G.C., Gonçalves M.C.P., Romanelli J.P., Lopes L.A., Berenguer-Murcia Á., Fernandez-Lafuente R., Tardioli P.W. (2023). A review of lipase immobilization on hydrophobic supports incorporating systematic mapping principles. React. Chem. Eng..

[60] Sampaio C.S., Angelotti J.A., Fernandez-Lafuente R., Hirata D.B. (2022). Lipase immobilization via cross-linked enzyme aggregates: Problems and prospects – A review. Int. J. Biol. Macromol..

[61] Wang N., Wang W., Su Y., Zhang J., Sun B., Ai N. (2025). The current research status of immobilized lipase performance and its potential for application in food are developing toward green and healthy direction: A review. J. Food Sci..

[62] Lee S.H., Doan T.T.N., Won K., Ha S.H., Koo Y.-M. (2010). Immobilization of lipase within carbon nanotube–silica composites for non-aqueous reaction systems. J. Mol. Catal. B Enzym..

[63] Chandra K., Dong C.-D., Chauhan A.S., Chen C.-W., Patel A.K., Singhania R.R. (2025). Advancements in lipase immobilization: Enhancing enzyme efficiency with nanomaterials for industrial applications. Int. J. Biol. Macromol..

[64] Handayani N., Loos K., Wahyuningrum D., Buchari, Zulfikar M.A. (2012). Immobilization of Mucor miehei Lipase onto Macroporous Aminated Polyethersulfone Membrane for Enzymatic Reactions. Membranes.

[65] Jasińska K., Nowosad M., Perzyna A., Bielacki A., Dziwiński S., Zieniuk B., Fabiszewska A. (2024). Sustainable Lipase Immobilization: Chokeberry and Apple Waste as Carriers. Biomolecules.

[66] Putra S.S.S., Chew C.Y., Hayyan A., Elgharbawy A.A., Taskin-Tok T., Hayyan M., Ngoh G.C., Saleh J., Al Abdulmonem W., Alghsham R.S. (2024). Nanodiamonds and natural deep eutectic solvents as potential carriers for lipase. Int. J. Biol. Macromol..

[67] Guajardo N., Ahumada K., de María P.D. (2020). Immobilized lipase-CLEA aggregates encapsulated in lentikats® as robust biocatalysts for continuous processes in deep eutectic solvents. J. Biotechnol..

[68] Guajardo N., Ahumada K., de María P.D. (2021). Immobilization of Pseudomonas stutzeri lipase through Cross-linking Aggregates (CLEA) for reactions in Deep Eutectic Solvents. J. Biotechnol..

[69] Bellou M.G., Gkantzou E., Skonta A., Moschovas D., Spyrou K., Avgeropoulos A., Gournis D., Stamatis H. (2022). Development of 3D Printed Enzymatic Microreactors for Lipase-Catalyzed Reactions in Deep Eutectic Solvent-Based Media. Micromachines.

[70] Buzatu A.R., Todea A., Pop R., Dreavă D.M., Paul C., Bîtcan I., Motoc M., Peter F., Boeriu C.G. (2025). Designed Reactive Natural Deep Eutectic Solvents for Lipase-Catalyzed Esterification. Molecules.

[71] Martínez-Baquero R., Martínez-Tomé M.J., Gómez J., Esquembre R., Mateo C.R. (2025). Pancreatic Lipase in Eutectogels as Emerging Materials: Exploring Their Properties and Potential Applications in Biosensing. Biosensors.

[72] Santos J., Dencheva N.V., Denchev Z.Z., Venâncio A., Silva C. (2026). Immobilization of Porcine Pancreatic Lipase onto Polyamide 6 for Selective Degradation of Ochratoxin A in Grape Pomace. Food Bioprocess Technol..

[73] Rueda N., dos Santos J.C.S., Ortiz C., Torres R., Barbosa O., Rodrigues R.C., Berenguer-Murcia Á., Fernandez-Lafuente R. (2016). Chemical Modification in the Design of Immobilized Enzyme Biocatalysts: Drawbacks and Opportunities. Chem. Rec..

[74] Noro J., Castro T.G., Cavaco-Paulo A., Silva C. (2020). Substrate hydrophobicity and enzyme modifiers play a major role in the activity of lipase from *Thermomyces lanuginosus*. Catal. Sci. Technol..

[75] Jia R., Hu Y., Liu L., Jiang L., Huang H. (2013). Chemical modification for improving activity and stability of lipase B from Candida antarctica with imidazolium-functional ionic liquids. Org. Biomol. Chem..

[76] Jia R., Hu Y., Liu L., Jiang L., Zou B., Huang H. (2013). Enhancing Catalytic Performance of Porcine Pancreatic Lipase by Covalent Modification Using Functional Ionic Liquids. ACS Catal..

[77] Xu C., Yin X., Zhang C., Chen H., Huang H., Hu Y. (2018). Improving Catalytic Performance of Burkholderiacepacia Lipase by Chemical Modification with Functional Ionic Liquids. Chem. Res. Chin. Univ..

[78] Li X., Zhang C., Li S., Huang H., Hu Y. (2015). Improving Catalytic Performance of *Candida rugosa* Lipase by Chemical Modification with Polyethylene Glycol Functional Ionic Liquids. Ind. Eng. Chem. Res..

[79] Jayawardena M.B., Yee L.H., Poljak A., Cavicchioli R., Kjelleberg S.J., Siddiqui K.S. (2017). Enhancement of lipase stability and productivity through chemical modification and its application to latex-based polymer emulsions. Process Biochem..

[80] Noro J., Cavaco-Paulo A., Silva C. (2021). Chemically Modified Lipase from *Thermomyces lanuginosus* with Enhanced Esterification and Transesterification Activities. ChemCatChem.

[81] Dozier J.K., Distefano M.D. (2015). Site-Specific PEGylation of Therapeutic Proteins. Int. J. Mol. Sci..

[82] Noro J., Castro T.G., Gonçalves F., Ribeiro A., Cavaco-Paulo A., Silva C. (2019). Catalytic Activation of Esterases by PEGylation for Polyester Synthesis. ChemCatChem.

[83] Zhang Y., Dai Y., Hou M., Li T., Ge J., Liu Z. (2013). Chemo-enzymatic synthesis of valrubicin using Pluronic conjugated lipase with temperature responsiveness in organic media. RSC Adv..

[84] Wu X., Wang R., Zhang Y., Ge J., Liu Z. (2014). Enantioselective Ammonolysis of Phenylglycine Methyl Ester with Lipase–Pluronic Nanoconjugate in Tertiary Butanol. Catal. Lett..

[85] Hou M., Wang R., Wu X., Zhang Y., Ge J., Liu Z. (2015). Synthesis of Lutein Esters by Using a Reusable Lipase-Pluronic Conjugate as the Catalyst. Catal. Lett..

[86] Kübelbeck S., Mikhael J., Keller H., Konradi R., Andrieu-Brunsen A., Baier G. (2018). Enzyme–Polymer Conjugates to Enhance Enzyme Shelf Life in a Liquid Detergent Formulation. Macromol. Biosci..

[87] Kajiwara S., Komatsu K., Yamada R., Matsumoto T., Yasuda M., Ogino H. (2019). Modification of lipase from Candida cylindracea with dextran using the borane-pyridine complex to improve organic solvent stability. J. Biotechnol..

[88] Kajiwara S., Komatsu K., Yamada R., Matsumoto T., Yasuda M., Ogino H. (2019). Improvement of the organic solvent stability of a commercial lipase by chemical modification with dextran. Biochem. Eng. J..

[89] Chado G.R., Holland E.N., Tice A.K., Stoykovich M.P., Kaar J.L. (2018). Modification of Lipase with Poly(4-acryloylmorpholine) Enhances Solubility and Transesterification Activity in Anhydrous Ionic Liquids. Biomacromolecules.

[90] Kovaliov M., Allegrezza M.L., Richter B., Konkolewicz D., Averick S. (2018). Synthesis of lipase polymer hybrids with retained or enhanced activity using the grafting-from strategy. Polymer.

[91] Chen N., Zhang C., Dong X., Liu Y., Sun Y. (2020). Activation and stabilization of lipase by grafting copolymer of hydrophobic and zwitterionic monomers onto the enzyme. Biochem. Eng. J..

[92] Spinei M., Oroian M. (2021). The Potential of Grape Pomace Varieties as a Dietary Source of Pectic Substances. Foods.

[93] Mandache M.B., Stoenescu A.-M., Cosmulescu S. (2024). Influence of Solvent Concentration on the Amount of Individual Phenolic Compounds in Apple and Sour Cherry Pomace. Horticulturae.

[94] Mondani L., Palumbo R., Tsitsigiannis D., Perdikis D., Mazzoni E., Battilani P. (2020). Pest Management and Ochratoxin A Contamination in Grapes: A Review. Toxins.

[95] Gonçalves A., Palumbo R., Guimarães A., Gkrillas A., Dall’aSta C., Dorne J.-L., Battilani P., Venâncio A. (2019). The Route of Mycotoxins in the Grape Food Chain. Am. J. Enol. Vitic..

[96] Solfrizzo M., Panzarini G., Visconti A. (2008). Determination of Ochratoxin A in Grapes, Dried Vine Fruits, and Winery Byproducts by High-Performance Liquid Chromatography with Fluorometric Detection (HPLC−FLD) and Immunoaffinity Cleanup. J. Agric. Food Chem..

[97] (2016). Commission Recommendation of 17 August 2006 on the Presence of Deoxynivalenol, Zearalenone, Ochratoxin A, T-2 and HT-2 and Fumonisins in Products Intended for Animal Feeding (2006/576/EC). Off. J. Eur. Union.

[98] Ribeiro E., Alves A. (2008). Comparative study of screening methodologies for ochratoxin A detection in winery by-products. Anal. Bioanal. Chem..

[99] Panić M., Gunjević V., Cravotto G., Redovniković I.R. (2019). Enabling technologies for the extraction of grape-pomace anthocyanins using natural deep eutectic solvents in up-to-half-litre batches extraction of grape-pomace anthocyanins using NADES. Food Chem..

[100] Punzo A., Porru E., Silla A., Simoni P., Galletti P., Roda A., Tagliavini E., Samorì C., Caliceti C. (2021). Grape Pomace for Topical Application: Green NaDES Sustainable Extraction, Skin Permeation Studies, Antioxidant and Anti-Inflammatory Activities Characterization in 3D Human Keratinocytes. Biomolecules.

[101] Vorobyova V., Skiba M., Horodniuk O., Khrokalo L., Vasyliev G. (2023). Betaine-Based Deep Eutectic Grape Pomace Extract Mediated Synthesis of Silver Nanoparticles with Antibacterial Activities. BioNanoScience.

[102] Li Z., Cui R., Liu W., Wang M., Li L., Liu F., Du B., Song L. (2024). Application of green deep eutectic solvents for anthocyanins extraction from grape pomace: Optimization, stability, antioxidant activity, and molecular dynamic simulation. LWT.

[103] Hocking A.D., Leong S.-L.L., Kazi B.A., Emmett R.W., Scott E.S. (2007). Fungi and mycotoxins in vineyards and grape products. Int. J. Food Microbiol..

[104] Jeong K.M., Zhao J., Jin Y., Heo S.R., Han S.Y., Yoo D.E., Lee J. (2015). Highly efficient extraction of anthocyanins from grape skin using deep eutectic solvents as green and tunable media. Arch. Pharmacal Res..

[105] Bubalo M.C., Ćurko N., Tomašević M., Ganić K.K., Redovniković I.R. (2016). Green extraction of grape skin phenolics by using deep eutectic solvents. Food Chem..

[106] Jara-Palacios M.J. (2019). Wine Lees as a Source of Antioxidant Compounds. Antioxidants.

[107] Kochman J., Jakubczyk K., Janda K. (2021). Mycotoxins in red wine: Occurrence and risk assessment. Food Control.

[108] Quintela S., Villarán M.C., de Armentia I.L., Elejalde E. (2013). Ochratoxin A removal in wine: A review. Food Control.

[109] Fernandes A., Ratola N., Cerdeira A., Alves A., Venâncio A. (2007). Changes in Ochratoxin A Concentration during Winemaking. Am. J. Enol. Vitic..

[110] La Placa L., Tsitsigiannis D., Leggieri M.C., Battilani P. (2023). From Grapes to Wine: Impact of the Vinification Process on Ochratoxin A Contamination. Foods.

[111] Pérez-Serradilla J., de Castro L. (2008). Role of lees in wine production: A review. Food Chem..

[112] Anli R.E., Vural N., Bayram M. (2011). Removal of Ochratoxin A (OTA) from Naturally Contaminated Wines During the Vinification Process. J. Inst. Brew..

[113] Bosiljkov T., Dujmić F., Bubalo M.C., Hribar J., Vidrih R., Brnčić M., Zlatic E., Redovniković I.R., Jokić S. (2017). Natural deep eutectic solvents and ultrasound-assisted extraction: Green approaches for extraction of wine lees anthocyanins. Food Bioprod. Process..

[114] Lyu F., Luiz S., Azeredo D.R., Cruz A., Ajlouni S., Ranadheera C.S. (2020). Apple Pomace as a Functional and Healthy Ingredient in Food Products: A Review. Processes.

[115] Yuan Y., Zhuang H., Zhang T., Liu J. (2010). Patulin content in apple products marketed in Northeast China. Food Control.

[116] Guo Y., Zhou Z., Yuan Y., Yue T. (2013). Survey of patulin in apple juice concentrates in Shaanxi (China) and its dietary intake. Food Control.

[117] Zhong L., Carere J., Lu Z., Lu F., Zhou T. (2018). Patulin in Apples and Apple-Based Food Products: The Burdens and the Mitigation Strategies. Toxins.

[118] Harris K.L., Bobe G., Bourquin L.D. (2009). Patulin Surveillance in Apple Cider and Juice Marketed in Michigan. J. Food Prot..

[119] Spadaro D., Ciavorella A., Frati S., Garibaldi A., Gullino M. (2007). Incidence and level of patulin contamination in pure and mixed apple juices marketed in Italy. Food Control.

[120] Kanetis L., Testempasis S., Goulas V., Samuel S., Myresiotis C., Karaoglanidis G.S. (2015). Identification and mycotoxigenic capacity of fungi associated with pre- and postharvest fruit rots of pomegranates in Greece and Cyprus. Int. J. Food Microbiol..

[121] Akl E.M., Embaby E.M., Younos M.A. (2024). Detection of Mycotoxigenic fungi associated with Pomegranate fruits (Punica granatum L.) and their control using Garden cress meal extract. Egypt. J. Chem..

[122] Hussain S., Asi M.R., Iqbal M., Khalid N., Wajih-Ul-Hassan S., Ariño A. (2020). Patulin Mycotoxin in Mango and Orange Fruits, Juices, Pulps, and Jams Marketed in Pakistan. Toxins.

[123] Shavakhi F., Rahmani A., Piravi-Vanak Z. (2022). A global systematic review and meta-analysis on prevalence of the aflatoxin B1 contamination in olive oil. J. Food Sci. Technol..

[124] Cavaliere C., Foglia P., Guarino C., Nazzari M., Samperi R., Laganà A. (2007). Determination of aflatoxins in olive oil by liquid chromatography–tandem mass spectrometry. Anal. Chim. Acta.

[125] Ferracane R., Tafuri A., Logieco A., Galvano F., Balzano D., Ritieni A. (2007). Simultaneous determination of aflatoxin B_1_and ochratoxin A and their natural occurrence in Mediterranean virgin olive oil. Food Addit. Contam..

[126] Gherbawy Y.A., Elhariry H.M., Bahobial A.A.S. (2012). Mycobiota and Mycotoxins (Aflatoxins and Ochratoxin) Associated with Some Saudi Date Palm Fruits. Foodborne Pathog. Dis..

[127] Azaiez I., Font G., Mañes J., Fernández-Franzón M. (2015). Survey of mycotoxins in dates and dried fruits from Tunisian and Spanish markets. Food Control.

[128] Abdallah M.F., Krska R., Sulyok M. (2018). Occurrence of Ochratoxins, Fumonisin B_2_, Aflatoxins (B_1_ and B_2_), and Other Secondary Fungal Metabolites in Dried Date Palm Fruits from Egypt: A Mini-Survey. J. Food Sci..

[129] Nikolchina I., Rodrigues P. (2021). A preliminary study on mycobiota and ochratoxin a contamination in commercial palm dates (Phoenix dactylifera). Mycotoxin Res..

[130] Daou R., Hoteit M., Bookari K., Joubrane K., Khabbaz L.R., Ismail A., Maroun R.G., el Khoury A. (2023). Public health risk associated with the co-occurrence of aflatoxin B1 and ochratoxin A in spices, herbs, and nuts in Lebanon. Front. Public Health.

[131] Garcia M.V., Mallmann C.A., Copetti M.V. (2018). Aflatoxigenic and ochratoxigenic fungi and their mycotoxins in spices marketed in Brazil. Food Res. Int..

[132] Santos L., Marín S., Sanchis V., Ramos A.J. (2009). Screening of mycotoxin multicontamination in medicinal and aromatic herbs sampled in Spain. J. Sci. Food Agric..

[133] García-Moraleja A., Font G., Mañes J., Ferrer E. (2015). Simultaneous determination of mycotoxin in commercial coffee. Food Control.

[134] Al-Abdalall A.H.A., Al-Thalib E.A. (2016). Aflatoxin and Ochratoxin Production in Ground Coffee During Storage. Can. J. Pure Appl. Sci..

[135] Mir M.A., Chang S.K., Ashraf M.W., Andrews K. (2024). Heavy metal and mycotoxin-producing fungi contamination of coffee consumed in Saudi Arabia. Food Chem. Adv..

[136] Mujahid C., Savoy M.-C., Baslé Q., Woo P.M., Ee E.C.Y., Mottier P., Bessaire T. (2020). Levels of *Alternaria* Toxins in Selected Food Commodities Including Green Coffee. Toxins.

[137] Hagos L., Guta M., Bacha K. (2024). Prevalence of mycotoxigenic fungi and ochratoxin A in coffee (*Coffea arabica* L.). Cogent Food Agric..

[138] Jalili M. (2016). Natural Occurrence of Ochratoxin A Contamination in Commercial Spices in Tehran. Nutr. Food Sci. Res..

[139] González L., Juan C., Soriano J., Moltó J., Mañes J. (2005). Occurrence and daily intake of ochratoxin A of organic and non-organic rice and rice products. Int. J. Food Microbiol..

[140] Li Y., Zhang X., Nie J., Bacha S.A.S., Yan Z., Gao G. (2020). Occurrence and co-occurrence of mycotoxins in apple and apple products from China. Food Control.

[141] Carbonell-Rozas L., Mihalache O.A., Dall’Asta C. (2025). Multi-class analysis of mycotoxin prevalence and potential health risks in commercial apple puree: Implications for early childhood populations in the Italian market. Food Res. Int..

[142] Ackermann Y., Curtui V., Dietrich R., Gross M., Latif H., Märtlbauer E., Usleber E. (2011). Widespread Occurrence of Low Levels of Alternariol in Apple and Tomato Products, as Determined by Comparative Immunochemical Assessment using Monoclonal and Polyclonal Antibodies. J. Agric. Food Chem..

[143] Carbonell-Rozas L., Albasi V., Leggieri M.C., Dall’aSta C., Battilani P. (2024). Apple mycotoxins: From orchard to processed apple puree. Fungal Biol..

[144] Ji X., Deng T., Xiao Y., Jin C., Lyu W., Wu Z., Wang W., Wang X., He Q., Yang H. (2022). Emerging Alternaria and Fusarium mycotoxins in tomatoes and derived tomato products from the China market: Occurrence, methods of determination, and risk evaluation. Food Control.

[145] Zhao K., Shao B., Yang D., Li F. (2014). Natural Occurrence of Four *Alternaria* Mycotoxins in Tomato- and Citrus-Based Foods in China. J. Agric. Food Chem..

[146] Santos G.G., Mattos L.M., Moretti C.L. (2016). Quality and Occurrence of Mycotoxins in Tomato Products in the Brazilian Market. Enzym. Eng..

[147] Sanzani S.M., Gallone T., Garganese F., Caruso A.G., Amenduni M., Ippolito A. (2019). Contamination of fresh and dried tomato by *Alternaria* toxins in southern Italy. Food Addit. Contam. Part A.

[148] Van de Perre E., Deschuyffeleer N., Jacxsens L., Vekeman F., Van Der Hauwaert W., Asam S., Rychlik M., Devlieghere F., De Meulenaer B. (2014). Screening of moulds and mycotoxins in tomatoes, bell peppers, onions, soft red fruits and derived tomato products. Food Control.

[149] Qiao X., Yin J., Yang Y., Zhang J., Shao B., Li H., Chen H. (2018). Determination of *Alternaria* Mycotoxins in Fresh Sweet Cherries and Cherry-Based Products: Method Validation and Occurrence. J. Agric. Food Chem..

[150] Juan C., Oueslati S., Mañes J. (2016). Evaluation of *Alternaria* mycotoxins in strawberries: Quantification and storage condition. Food Addit. Contam. Part A.

[151] Kłapeć T., Wójcik-Fatla A., Farian E., Kowalczyk K., Cholewa G., Cholewa A., Dutkiewicz J. (2022). Mycobiota of berry fruits – levels of filamentous fungi and mycotoxins, composition of fungi, and analysis of potential health risk for consumers. Ann. Agric. Environ. Med..

[152] Kłapeć T., Wójcik-Fatla A., Farian E., Kowalczyk K., Cholewa G., Cholewa A., Dutkiewicz J. (2022). Mycobiota and mycotoxins in various kinds of vegetables and fruits as potential health risk factors for consumers – summary of a multiyear study. Ann. Agric. Environ. Med..

[153] Barboráková Z., Jakabova S., Maskova Z., Mrvova M., Uzsákova V., Makova J., Tancinova D. (2023). Toxin producing micromycetes of the genus penicillium and aspergillus on berries, grapes, and tomato fruits in slovak stores. J. Microbiol. Biotechnol. Food Sci..

[154] Varga J., Kocsubé S., Szigeti G., Man V., Tóth B., Vágvölgyi C., Bartók T. (2012). Black Aspergilli and fumonisin contamination in onions purchased in Hungary. Acta Aliment..

[155] Rämö S., Haapalainen M., Latvala S. (2021). Development and Validation of a UHPLC-MS/MS Method for the Analysis of Fusarium Mycotoxins in Onion. Food Anal. Methods.

[156] Škrbić B., Živančev J., Đurišić-Mladenović N., Godula M. (2012). Principal mycotoxins in wheat flour from the Serbian market: Levels and assessment of the exposure by wheat-based products. Food Control.

[157] dos Santos I.D., Pizzutti I.R., Dias J.V., Fontana M.E.Z., Souza D.M., Cardoso C.D. (2021). Mycotoxins in wheat flour: Occurrence and co-occurrence assessment in samples from Southern Brazil. Food Addit. Contam. Part B.

[158] Zhu Z., Guo W., Cheng H., Zhao H., Wang J., Abdallah M.F., Zhou X., Lei H., Tu W., Wang H. (2024). Co-contamination and interactions of multiple mycotoxins and heavy metals in rice, maize, soybeans, and wheat flour marketed in Shanghai City. J. Hazard. Mater..

[159] Kołodziejczyk K., Sójka M., Abadias M., Viñas I., Guyot S., Baron A. (2013). Polyphenol composition, antioxidant capacity, and antimicrobial activity of the extracts obtained from industrial sour cherry pomace. Ind. Crop. Prod..

[160] Vázquez-González M., Fernández-Prior Á., Oria A.B., Rodríguez-Juan E.M., Pérez-Rubio A.G., Fernández-Bolaños J., Rodríguez-Gutiérrez G. (2020). Utilization of strawberry and raspberry waste for the extraction of bioactive compounds by deep eutectic solvents. LWT.

[161] Bottu H.M., Mero A., Husanu E., Tavernier S., Pomelli C.S., Dewaele A., Bernaert N., Guazzelli L., Brennan L. (2022). The ability of deep eutectic solvent systems to extract bioactive compounds from apple pomace. Food Chem..

[162] Rashid R., Wani S.M., Manzoor S., Masoodi F., Dar M.M. (2022). Green extraction of bioactive compounds from apple pomace by ultrasound assisted natural deep eutectic solvent extraction: Optimisation, comparison and bioactivity. Food Chem..

[163] Wang H., Deng M., Zhang Z., Zhang J., Wu K., Feng J., Li S., Tong Y.W. (2025). Ultrasound-assisted extraction of polyphenols from apple pomace using choline chloride-oxalic acid deep eutectic solvent: Characterization, reusability, and molecular dynamics simulation. Microchem. J..

[164] Popovic B.M., Micic N., Potkonjak A., Blagojevic B., Pavlovic K., Milanov D., Juric T. (2022). Novel extraction of polyphenols from sour cherry pomace using natural deep eutectic solvents – Ultrafast microwave-assisted NADES preparation and extraction. Food Chem..

[165] Benítez-Correa E., Bastías-Montes J.M., Acuña-Nelson S., Muñoz-Fariña O. (2023). Effect of choline chloride-based deep eutectic solvents on polyphenols extraction from cocoa (Theobroma cacao L.) bean shells and antioxidant activity of extracts. Curr. Res. Food Sci..

[166] Garofalo S.F., Mallen V., Fino D. (2025). Extraction of carotenoids from tomato pomace using deep eutectic solvents composed of short and medium-chain fatty acids and menthol. Food Chem..

[167] Lazzarini C., Casadei E., Valli E., Tura M., Ragni L., Bendini A., Toschi T.G. (2022). Sustainable Drying and Green Deep Eutectic Extraction of Carotenoids from Tomato Pomace. Foods.

[168] Vorobyova V., Skiba M., Vasyliev G. (2021). Extraction of phenolic compounds from tomato pomace using choline chloride–based deep eutectic solvents. J. Food Meas. Charact..

[169] Vasyliev G., Lyudmyla K., Hladun K., Skiba M., Vorobyova V. (2022). Valorization of tomato pomace: Extraction of value-added components by deep eutectic solvents and their application in the formulation of cosmetic emulsions. Biomass Convers. Biorefinery.

[170] Wawoczny A., Wilk J., Shyntum D., Shakibania S., Krukiewicz K., Gibas J., Machulik M., Płonka J., Bajkacz S., Dudek G. (2025). Valorization of waste tomato leaves with natural deep eutectic solvents. Food Chem..

[171] Bertolo M.R., Oliveira L.F.R., Titato G.M., Lanças F.M., Correa D.S. (2025). Sustainable extraction of value-added compounds from orange waste using natural deep eutectic solvents. J. Mol. Liq..

[172] Gomez-Urios C., Siroli L., Grassi S., Patrignani F., Blesa J., Lanciotti R., Frígola A., Iametti S., Esteve M.J., Di Nunzio M. (2025). Sustainable valorization of citrus by-products: natural deep eutectic solvents for bioactive extraction and biological applications of Citrus sinensis peel. Eur. Food Res. Technol..

[173] Domínguez-Rodríguez G., Amador-Luna V.M., Castro-Puyana M., Ibáñez E., Marina M.L. (2025). Sustainable strategies to obtain bioactive compounds from citrus peels by supercritical fluid extraction, ultrasound-assisted extraction, and natural deep eutectic solvents. Food Res. Int..

[174] El Kantar S., Rajha H.N., Boussetta N., Vorobiev E., Maroun R.G., Louka N. (2019). Green extraction of polyphenols from grapefruit peels using high voltage electrical discharges, deep eutectic solvents and aqueous glycerol. Food Chem..

[175] Hernández-Corroto E., Plaza M., Marina M.L., García M.C. (2020). Sustainable extraction of proteins and bioactive substances from pomegranate peel (Punica granatum L.) using pressurized liquids and deep eutectic solvents. Innov. Food Sci. Emerg. Technol..

[176] Chanioti S., Tzia C. (2018). Extraction of phenolic compounds from olive pomace by using natural deep eutectic solvents and innovative extraction techniques. Innov. Food Sci. Emerg. Technol..

[177] Panić M., Stojković M.R., Kraljić K., Škevin D., Redovniković I.R., Srček V.G., Radošević K. (2019). Ready-to-use green polyphenolic extracts from food by-products. Food Chem..

[178] Fernández M.d.L.Á., Espino M., Gomez F.J., Silva M.F. (2018). Novel approaches mediated by tailor-made green solvents for the extraction of phenolic compounds from agro-food industrial by-products. Food Chem..

[179] Alañón M., Ivanović M., Gómez-Caravaca A., Arráez-Román D., Segura-Carretero A. (2020). Choline chloride derivative-based deep eutectic liquids as novel green alternative solvents for extraction of phenolic compounds from olive leaf. Arab. J. Chem..

[180] García A., Rodriguez-Juan E., Rodriguez-Gutiérrez G., Rios J.J., Fernández-Bolaños J. (2016). Extraction of phenolic compounds from virgin olive oil by deep eutectic solvents (DESs). Food Chem..

[181] Rodríguez-Juan E., Rodríguez-Romero C., Fernández-Bolaños J., Florido M.C., Garcia-Borrego A. (2020). Phenolic compounds from virgin olive oil obtained by natural deep eutectic solvent (NADES): Effect of the extraction and recovery conditions. J. Food Sci. Technol..

[182] Pal C.B.T., Jadeja G.C. (2018). Deep eutectic solvent-based extraction of polyphenolic antioxidants from onion (*Allium cepa* L.) peel. J. Sci. Food Agric..

[183] Gullón B., Muñiz-Mouro A., Lú-Chau T.A., Moreira M.T., Lema J.M., Eibes G. (2019). Green approaches for the extraction of antioxidants from eucalyptus leaves. Ind. Crop. Prod..

[184] Zeng J., Dou Y., Yan N., Li N., Zhang H., Tan J.-N. (2019). Optimizing Ultrasound-Assisted Deep Eutectic Solvent Extraction of Bioactive Compounds from Chinese Wild Rice. Molecules.

[185] Airouyuwa J.O., Mostafa H., Riaz A., Stathopoulos C., Maqsood S. (2022). Natural Deep Eutectic Solvents and Microwave-Assisted Green Extraction for Efficient Recovery of Bioactive Compounds from By-Products of Date Fruit (Phoenix dactylifera L.) Processing: Modeling, Optimization, and Phenolic Characterization. Food Bioprocess Technol..

[186] Loukri A., Sarafera C., Goula A.M., Gardikis K., Mourtzinos I. (2022). Green extraction of caffeine from coffee pulp using a deep eutectic solvent (DES). Appl. Food Res..

[187] Fanali C., Della Posta S., Dugo L., Gentili A., Mondello L., De Gara L. (2020). Choline-chloride and betaine-based deep eutectic solvents for green extraction of nutraceutical compounds from spent coffee ground. J. Pharm. Biomed. Anal..

[188] Khezeli T., Daneshfar A., Sahraei R. (2016). A green ultrasonic-assisted liquid–liquid microextraction based on deep eutectic solvent for the HPLC-UV determination of ferulic, caffeic and cinnamic acid from olive, almond, sesame and cinnamon oil. Talanta.

[189] Zhang R., Chen H., Yu Q., Zhang Y., Liu F., Wang F., Chen X., Liu Y. (2023). Extraction of bioactive compounds from cinnamon residues with deep eutectic solvents and its molecular mechanism. Chem. Eng. Sci..

[190] Faraji M., Mahmoodi-Maymand M., Dastmalchi F. (2020). Green, fast and simple dispersive liquid-liquid microextraction method by using hydrophobic deep eutectic solvent for analysis of folic acid in fortified flour samples before liquid chromatography determination. Food Chem..

